# State and Fault Estimation for Uncertain Complex Networks Using Binary Encoding Schemes Under Switching Couplings and Deception Attacks

**DOI:** 10.3390/s26010182

**Published:** 2025-12-26

**Authors:** Nan Hou, Mengdi Chang, Hongyu Gao, Zhongrui Hu, Xianye Bu

**Affiliations:** 1 Sanya Offshore Oil & Gas Research Institute of Northeast Petroleum University, Sanya 572025, China; nanhou@nepu.edu.cn (N.H.); 071979020102@nepu.edu.cn (H.G.); zhong_rui_hu@nepu.edu.cn (Z.H.); 2 Artificial Intelligence Energy Research Institute, Northeast Petroleum University, Daqing 163318, China; 218001060086@stu.nepu.edu.cn; 3 State Key Laboratory of Continental Shale Oil, Northeast Petroleum University, Daqing 163318, China; 4 Heilongjiang Provincial Key Laboratory of Networking and Intelligent Control, Northeast Petroleum University, Daqing 163318, China; 5 Research Center for Mathematics and Interdisciplinary Sciences, Northeast Petroleum University, Daqing 163318, China; 6 School of Electrical & Information Engineering, Northeast Petroleum University, Daqing 163318, China

**Keywords:** binary encoding schemes, state and fault estimation, randomly switching couplings, complex networks, deception attacks

## Abstract

A state and fault estimator is designed in this paper for nonlinear complex networks using binary encoding schemes subject to parameter uncertainties, randomly switching couplings, randomly occurring deception attacks and bounded stochastic noises. A Markov chain is employed to reflect the randomly switching phenomena of topological structures (or outer coupling strengths) and internal coupling strengths in complex networks. Binary encoding scheme is utilized to adjust the measurement signal transmission, where the signal is quantized and encoded into a binary bit string which is transmitted via a binary symmetric channel. Random bit flipping resulted from channel noises and randomly occurring deception attacks launched by hacker may take place inevitably during the network transmission process, whose occurrences are represented by two sequences of Bernoulli distributed random variables. The influence of random bit flipping is viewed as an equivalent stochastic noise, which facilitates the estimator design afterwards. The malicious signal is characterized by a nonlinear function satisfying an inequality constraint condition. The received binary bit string is decoded and used for estimating the system state and the fault. This paper aims to design a state and fault estimator such that the estimation error dynamic system is exponentially ultimately bounded in mean square, and the ultimate upper bound is minimized. A sufficient condition is put forth that ensures the existence of the expected state and fault estimator via adopting statistical property analysis, Lyapunov stability theory and matrix inequality technique. An exponentially ultimately bounded state and fault estimator in mean square is designed for such a kind of complex networks using the matrix inequality method. The estimator gain parameter is readily obtained by tackling an optimization issue subject to matrix inequalities constraints using Matlab software. Finally, two simulation examples are carried on which validate the effectiveness of the proposed state and fault estimation approach. The work in this paper plays a role in enriching the research system of estimation for complex network, and providing theoretical guidance for engineering applications.

## 1. Introduction

Complex networks (CNs) are familiar to researchers in multiple areas such as mathematics, computer science, control and geology, which comprise substantial nodes and couplings among or inside nodes [1,2,3]. In [4], CNs have been employed to model and study microfracture activity in rock mechanics. Estimation for CN is to acquire the state information of network nodes, which has attracted many researchers’ attention [5,6,7,8]. For example, in [8], set-membership state estimation has been realized for multirate nonlinear CNs under FlexRay protocols using a neural network-based method. In similar to the demand of grasping system state information, system fault information is also of major concern due to that fault may occur inevitably as a result of human misconduct or daily wastage [9,10,11,12,13]. Recognizing fault in a timely manner is crucial to prohibit damages to operation of the whole system, which is particularly true for CN systems with interactions among coupled network nodes [14,15]. Fault estimation is able to attain the size of the fault signal, which is conducive for fault-tolerant control [16]. Hence, the study of state and fault estimation issue for CNs is of significance and necessity.

As a research trend, system security issue has been put more and more emphasis on in various areas including financial industry and industrial manufacturing, which targets at weakening negative influence from hackers to a minimum degree [17,18,19,20]. With the quick development of open network in information era, cyber attacks may occur unavoidably posing serious threat to system stability [21,22,23]. As a common type of cyber attacks, deception attacks have attracted researchers’ interest which have been formulated mathematically in different models, such as the entire replacement of the original transmission signal by (1) a norm-bounded signal in [24], (2) a nonlinear function with Lipschitz condition in [25], and (3) a signal with bounded distinctions from both the original transmission signal and the previous received signal in [26]. In particular, the distributed state estimator has been designed in [24] for CNs suffering from deception attacks from the defender’s perspective, and the uniform mean square boundedness has been maintained of the derived covariance upper bound. Nevertheless, the security-guaranteed state and fault estimation problem for CNs has not been sufficiently addressed, which inspires the work of this paper.

Note that a critical characteristic of CNs is coupling (the linking strength) which contains the outer coupling among two network nodes and the inner coupling inside a single node [27,28,29]. In fact, the coupling may not be invariant caused by working condition changes (e.g., temperature and humidity) or unpredictable disturbances (e.g., power outage and element aging) [30,31,32]. For the Internet integrating interconnected websites, when a website server breaks down abruptly, its links with other websites lose effectiveness (the corresponding coupling becomes zero); and when the website server begins to work, its links with other websites recover (the corresponding coupling exists). In recent decades, stochastic couplings have been discussed which are described mathematically by using a Bernoulli-distributed random variable (i.e., two types of couplings), a scalar Wiener process and the combination of a Markov chain and the Kronecker delta function. For instance, stochastic coupling has been taken account of in [31] to reflect the discrete and random essence of network interaction topology. The design of estimators which are capable of resisting stochastic couplings in CNs is consistent with fact and of research value.

Nowadays, digitalized networked control systems are intrinsically discrete-time ones, and much research effort has been put into the analysis and synthesis issues of discrete-time systems [33,34,35]. In order to reduce the network bandwidth consumption and develop the information security during the network transmission process, a series of communication schemes has been widely employed to transmit signal (e.g., event-triggered mechanisms, communication protocols and encoding-decoding mechanisms) [36,37,38,39,40]. Recently, binary encoding schemes (BESs) have been often selected as the transmission means, where the transmitted code is the binary bit string (BBS) obtained by quantization and encoding [41]. In particular, random bit flipping may appear owing to the negative effect from channel noises, which would cause a difference between the decoded signal and the quantized signal. Several results have been found on dealing with random bit flipping, and effective methods have been proposed to guarantee the system performance against the signal transmission error [42,43]. Noting that few results have been yielded on the aspect of fault estimation for CN suffering from deception attacks and stochastic couplings, needless to mention the situation that BESs are adopted simultaneously, and this also inspires the work of this paper.

Most real networked control systems may exhibit complicated characteristics intrinsically, which include nonlinearity as a result of possible elements property change depending on working environment [44,45,46,47,48]. In particular, each network node of a CN is usually seen as a nonlinear subsystem. In [30], nonlinearity has been taken account of in the CN with a sector-bounded constraint, and the secure state estimation has been achieved against replay attacks. Also, systems may be interrupted by stochastic noises due to the fact that outer disturbances are unpredicted and not fixed [49,50,51,52]. For instance, the flight transportation network may be disturbed by bad weather or temporary military exercises, and the power network may be perturbed by burden variation or temperature lift. Parameter uncertainties may occur unavoidably in networked control systems caused by amplitude modulation or roundoff errors [53,54,55], which have been modelled by using multiplicative noises [56], the combination of a Gaussian white noise sequence and a constrained unknown nonlinear function [57], a variable varying within a given interval [30], and a norm-bounded form [58]. In consideration of approaching the reality and characterizing accurately, a CN model is rational if nonlinearity, stochastic noises and parameter uncertainties are included.

In summary, this paper estimates the state and fault for nonlinear CNs subject to uncertainties, randomly switching couplings (RSCs) and randomly occurring deception attacks (RODAs) using BESs. Difficulties to be tackled involve (1) how to characterize this state and fault estimation problem strictly within a unified framework; (2) how to reflect the impact from uncertainties, switching couplings, deception attacks and bit flippings; and (3) how to design the exponentially ultimately bounded state and fault estimator in mean square? *The main contributions of this paper are emphasized as follows: (1) the state and fault estimation task is accomplished for a comprehensive CN model which considers uncertainties, RSCs, RODAs and nonlinearity under BESs; (2) a sufficient condition is put forth for assuring the existence of the expected state and fault estimator, under which the performance constraint of exponential ultimate boundedness in mean square is satisfied of the estimation error dynamics (EED), and the ultimate upper bound is minimized; and (3) the gain parameter of the desired state and fault estimator is designed readily via solving the solution to an optimization issue constrained by certain matrix inequalities.*

The structure of this paper is outlined as follows. In Section 2, the system and estimator models and the performance requirement to be fulfilled are presented. In Section 3, the estimator design approach is developed after detailed analysis process, which guarantees that the EED achieves exponential ultimate boundedness in mean square with a minimized ultimate upper bound. In Section 4, two simulation examples are implemented, whose results verify the correctness of the developed estimation approach. In Section 5, the work done in this paper is summarized.

**Notation** **1.** *In this paper, the symbol is standard except stated especially. $R^{n}$ is n-dimensional Euclidean space. $R^{p\times q}$ means that the matrix dimension is p×q. tr(O), $O^{-1}$, $\lambda_{\min}\left(O\right)$ and $\lambda_{\max}\left(O\right)$ are the trace, the inverse, the minimum eigenvalue and the maximum eigenvalue of a square matrix O. Y<0 (or Y>0) and Y≤0 (or Y≥0) denote that Y is negative definite (or positive definite) and negative semi-definite (or positive semi-definite), respectively.* ⊗ *is the Kronecker product. ∥·∥ is the norm symbol. diag is on behalf of a block diagonal matrix. $B^{T}$ is the transpose of a matrix B. E{s} and V{s} manifest the expectation and the variance of a random variable or vector s. Prob{·} stands for the occurrence probability of the event “·”. $I_{\left(u\right)}$ indicates an identity matrix with a dimension u×u.*

## 2. Problem Formulation and Preliminaries

### 2.1. The Model of Network Nodes

Considering a CN system with *N* nodes:

(1) $$\begin{array}{cc} x_{i,k+1}= & \left(A_{i}+\Delta A_{i}\right)x_{i,k}+\left(H_{i}+\Delta H_{i}\right)h_{x_{i,k}}+E_{i}w_{k}+\sum_{j=1}^{N}p_{ij,q_{k}}\Gamma_{q_{k}}x_{j,k}+G_{i}f_{i,k} \end{array}$$

(2) $$\begin{array}{cc} y_{i,k}= & C_{i}x_{i,k},i=1,2,\ldots ,N \end{array}$$

where $x_{i,k}\in R^{o_{x}}$ and $y_{i,k}\in R^{o_{y}}$ represent the state vector and the measurement output signal of node *i*. $w_{k}\in R^{o_{w}}$ is the process noise described by a bounded stochastic noise sequence with $E\{w_{k}\}=0$ and $V\left\{w_{k}\right\}=w_{0}^{2}$. $A_{i}$, $H_{i}$, $E_{i}$, $G_{i}$ and $C_{i}$ are known parameter matrices with appropriate dimensions. The nonlinear vector function $h_{x_{i,k}}\in R^{o_{x}}$ ($h_{0}=0$) follows the constraint condition:

(3) $$\begin{array}{cc} {}[h_{u}-h_{v}- & \Psi_{1}{\left(u-v\right)]}^{T}\left[h_{u}-h_{v}-\Psi_{2}\left(u-v\right)\right]\leq 0 \end{array}$$

where $\Psi_{1}$ and $\Psi_{2}$ are given matrices. $P_{q_{k}}≜{\left[p_{ij,q_{k}}\right]}_{N\times N}$ is the external coupling configuration matrix that depicts the connections between nodes, where $p_{ij,q_{k}}\geq 0$ (i≠j), but not all are 0. Generally speaking, $P_{q_{k}}$ is a symmetric matrix and satisfies the following expression:

(4) $$\begin{array}{c} \sum_{j=1}^{N}p_{ij,q_{k}}=\sum_{j=1}^{N}p_{ji,q_{k}}=0,i=1,2,\ldots ,N. \end{array}$$

Markov chain $q_{k}$(k≥0) takes values in a finite state space {1,2,…,U}, and the elements in the transition probability matrix $Y≜{\left[\pi_{♭℘}\right]}_{U\times U}$ are given as follows:

(5) $$\begin{array}{c} \mathrm{Prob}\left\{q_{k+1}=℘|q_{k}=♭\right\}=\pi_{♭℘},\forall ♭,℘\in \left\{1,2,\ldots ,U\right\} \end{array}$$

where $\pi_{♭℘}$ means the transition probability from mode ♭ to mode *℘* with $\pi_{♭℘}⩾0$, and $\sum_{℘=1}^{U}\pi_{♭℘}=1$. $\Gamma_{q_{k}}≜\mathrm{diag}\left\{{ℵ}_{1,q_{k}},{ℵ}_{2,q_{k}},\ldots ,{ℵ}_{o_{x},q_{k}}\right\}\geq 0$ is the internal coupling matrix connecting the *r*th $\left(r=1,2,\ldots ,o_{x}\right)$ state variable if ${ℵ}_{r,q_{k}}\neq 0$.

**Remark** **1.** *Affected by factors such as environmental interference and component aging, the outer and inner connection strengths of network nodes may vary and exhibit random characteristics [31]. In this paper, a Markov chain $q_{k}$ is used to characterize and analyze the RSCs of the CN (1).*

$\Delta A_{i}$ and $\Delta H_{i}$ denote parameter uncertainties satisfying

(6) $$\begin{array}{c} \left[\begin{array}{cc} \Delta A_{i} & \Delta H_{i} \end{array}\right]=J_{1i}J_{2}\left[\begin{array}{cc} J_{3A} & J_{3H} \end{array}\right] \end{array}$$

where $J_{1i}$, $J_{3A}$ and $J_{3H}$ are given matrices with rational dimensions and the unknown matrix $J_{2}$ meets $J_{2}J_{2}^{T}\leq I$.

$f_{i,k}\in R^{o_{f}}$ is the node fault signal with

(7) $$\begin{array}{c} {\overset{˘}{f}}_{i,k+1}-{\overset{˘}{f}}_{i,k}=0 \end{array}$$

where ${\overset{˘}{f}}_{i,k}≜f_{i,k+1}-f_{i,k}$.

Defining ${\overset{˘}{x}}_{i,k}≜{\left[\begin{array}{ccc} x_{i,k}^{T} & f_{i,k}^{T} & {\overset{˘}{f}}_{i,k}^{T} \end{array}\right]}^{T}$, the following form is attained from (1), (2) and (7):

(8) $$\begin{array}{cc} {\overset{˘}{x}}_{i,k+1}= & \left({\overset{˘}{A}}_{i}+\Delta {\overset{˘}{A}}_{i}\right){\overset{˘}{x}}_{i,k}+\left({\overset{˘}{H}}_{i}+\Delta {\overset{˘}{H}}_{i}\right)h_{\overset{˘}{I}{\overset{˘}{x}}_{i,k}}+{\overset{˘}{E}}_{i}w_{k}+\sum_{j=1}^{N}p_{ij,q_{k}}{\overset{˘}{\Gamma }}_{q_{k}}{\overset{˘}{x}}_{j,k}\\ y_{i,k}= & {\overset{˘}{C}}_{i}{\overset{˘}{x}}_{i,k} \end{array}$$

where

$$\begin{array}{cc} \; & {\overset{˘}{A}}_{i}≜\left[\begin{array}{ccc} A_{i} & G_{i} & 0\\ 0 & I & I\\ 0 & 0 & I \end{array}\right],{\overset{˘}{H}}_{i}≜\left[\begin{array}{c} H_{i}\\ 0\\ 0 \end{array}\right],\Delta {\overset{˘}{H}}_{i}≜\left[\begin{array}{c} \Delta H_{i}\\ 0\\ 0 \end{array}\right],{\overset{˘}{E}}_{i}≜\left[\begin{array}{c} E_{i}\\ 0\\ 0 \end{array}\right],\\ \; & \Delta {\overset{˘}{A}}_{i}≜\mathrm{diag}\left\{\Delta A_{i},0,0\right\},\overset{˘}{I}≜\left[\begin{array}{ccc} I & 0 & 0 \end{array}\right],{\overset{˘}{\Gamma }}_{q_{k}}≜\mathrm{diag}\left\{\Gamma_{q_{k}},0,0\right\},{\overset{˘}{C}}_{i}≜\left[\begin{array}{ccc} C_{i} & 0 & 0 \end{array}\right]. \end{array}$$

### 2.2. The Signal Transmission Process

#### 2.2.1. BES

A BES is adopted to coordinate the measurement signal transmission. Considering that a finite bit budget is put into use actually to encode the signal through communication channels due to the limited communication bandwidth, and signals ought to be pretreated via quantization. Through quantizing and encoding the measurement signal $y_{i,k}$, a finite-length BBS is attained which is transmitted through a memoryless binary symmetric channel (BSC).

Suppose that the scope of a scalar signal $y_{il,k}$ (the *l*th component of $y_{i,k}$ with $l=1,2,\ldots ,o_{y}$) is [−L,L], where the scalar L∈R>0 depends on application. A binary encoder is adopted to convert $y_{il,k}$ into a BBS with length *S*. The whole interval [−L,L] is split into $2^{S}-1$ parts with the uniform interval length below:

(9) $$\begin{array}{c} \theta =2L/(2^{S}-1). \end{array}$$

Represent the uniformly located $2^{S}$ points (endpoints on both sides and spaced points inside) as

$$\begin{array}{c} O≜\{\ell^{\left(1\right)},\ell^{\left(2\right)},\ell^{\left(3\right)},\ldots ,\ell^{\left(2^{S}\right)}\} \end{array}$$

where $\ell^{\left(a\right)}≜-L+\left(a-1\right)\theta$ with $a=1,2,\ldots ,2^{S}$.

The following probabilistic quantization function $Q_{il,k}$ is utilized to quantize the signal $y_{il,k}$:

(10) $$\begin{array}{c} Q_{il,k}:y_{il,k}\to {\bar{y}}_{il,k} \end{array}$$

where ${\bar{y}}_{il,k}$ is the quantized signal. When $\ell^{\left(a\right)}\leq y_{il,k}\leq \ell^{\left(a+1\right)}$, the signal ${\bar{y}}_{il,k}$ is determined according to the following probabilistic manner:

(11) $$\begin{array}{cc} \; & \mathrm{Prob}\left\{{\bar{y}}_{il,k}=\ell^{\left(a\right)}\right\}=1-{\bar{\delta }}_{il,k},\mathrm{Prob}\left\{{\bar{y}}_{il,k}=\ell^{\left(a+1\right)}\right\}={\bar{\delta }}_{il,k} \end{array}$$

where ${\bar{\delta }}_{il,k}≜\frac{y_{il,k}-\ell^{\left(a\right)}}{\theta }$ and $0\leq {\bar{\delta }}_{il,k}\leq 1$. Note that what we obtain is the probability of ${\bar{y}}_{il,k}=\ell^{\left(a\right)}$ (or ${\bar{y}}_{il,k}=\ell^{\left(a+1\right)}$), which depends on $y_{il,k}$.

Set

(12) $$\begin{array}{c} n_{il,k}≜{\bar{y}}_{il,k}-y_{il,k} \end{array}$$

as the quantization error. In terms of (11), one has that $n_{il,k}$ is a random variable conforming to the Bernoulli distribution as follows:

(13) $$\begin{array}{cc} \; & \mathrm{Prob}\left\{n_{il,k}=-{\bar{\delta }}_{il,k}\theta \right\}=1-{\bar{\delta }}_{il,k},\mathrm{Prob}\left\{n_{il,k}=\left(1-{\bar{\delta }}_{il,k}\right)\theta \right\}={\bar{\delta }}_{il,k}. \end{array}$$

We get from (13) that

(14) $$\begin{array}{c} E\left\{n_{il,k}\right\}=0,V\left\{n_{il,k}\right\}={\overset{ˇ}{\delta }}_{il,k}\leq \theta^{2}/4 \end{array}$$

where ${\overset{ˇ}{\delta }}_{il,k}≜\theta^{2}{\bar{\delta }}_{il,k}\left(1-{\bar{\delta }}_{il,k}\right)$. Due to the fact that quantization is operated independently of $y_{il,k}$, we have $n_{il,k}$ are mutually independent.

An encoding function $M_{il,k}$ is resorted to which transforms the quantized signal ${\bar{y}}_{il,k}$ into the following BBSs:

(15) $$\begin{array}{c} M_{il,k}:{\bar{y}}_{il,k}\to B_{il,k} \end{array}$$

where $B_{il,k}≜\left\{\beta_{il,k}^{\left(1\right)},\beta_{il,k}^{\left(2\right)},\ldots ,\beta_{il,k}^{\left(S\right)}\right\}$ ($\beta_{il,k}^{\left(\epsilon \right)}\in \left\{0,1\right\},\epsilon =1,2,\ldots ,S$) denotes the BBS which is attained via

(16) $$\begin{array}{c} {\bar{y}}_{il,k}=-L+\sum_{\epsilon =1}^{S}\beta_{il,k}^{\left(\epsilon \right)}2^{\epsilon -1}\theta . \end{array}$$

Passing the transformation, the BBS $B_{il,k}$ is then sent through a memoryless BSC where each bit may flip caused by channel noises, and the flipping probability is called crossover probability. Subsequently, the received BBS is expressed as

$$\begin{array}{cc} D_{il,k}≜ & \left\{\zeta_{il,k}^{\left(1\right)},\zeta_{il,k}^{\left(2\right)},\ldots ,\zeta_{il,k}^{\left(S\right)}\right\},\zeta_{il,k}^{\left(\epsilon \right)}\in \left\{0,1\right\},\epsilon =1,2,\ldots ,S \end{array}$$

where $\zeta_{il,k}^{\left(\epsilon \right)}=\mu_{il,k}^{\left(\epsilon \right)}\left(1-\beta_{il,k}^{\left(\epsilon \right)}\right)+\left(1-\mu_{il,k}^{\left(\epsilon \right)}\right)\beta_{il,k}^{\left(\epsilon \right)}$ with

(17) $$\begin{array}{c} \mu_{il,k}^{\left(\epsilon \right)}=\left\{\begin{array}{c} 1,\;\mathrm{the}\;\epsilon \text{-}\mathrm{th}\mathrm{bit}\mathrm{flips}\\ 0,\;\mathrm{the}\;\epsilon \text{-}\mathrm{th}\mathrm{bit}\mathrm{does}\mathrm{not}\mathrm{flip}. \end{array}\right. \end{array}$$

The probabilities of $\mu_{il,k}^{\left(\epsilon \right)}$ are illustrated as follows:

$$\begin{array}{cc} \; & \mathrm{Prob}\left\{\mu_{il,k}^{\left(\epsilon \right)}=1\right\}=\bar{\mu },\mathrm{Prob}\left\{\mu_{il,k}^{\left(\epsilon \right)}=0\right\}=1-\bar{\mu } \end{array}$$

where $\bar{\mu }\in \left[0,1\right]$ represents the crossover probability. Assume that $\mu_{il,k}^{\left(\epsilon \right)}$(ϵ=1,2,…,S) are mutually independent and identically distributed.

When decoding the attained BBS $D_{il,k}$, a decoding function $T_{il,k}$ is adopted as below:

(18) $$\begin{array}{c} T_{il,k}:D_{il,k}\to {\tilde{y}}_{il,k} \end{array}$$

where ${\tilde{y}}_{il,k}$ is the restored signal after network transmission whose expression is as follows:

(19) $$\begin{array}{c} {\tilde{y}}_{il,k}=-L+\sum_{\epsilon =1}^{S}\zeta_{il,k}^{\left(\epsilon \right)}2^{\epsilon -1}\theta . \end{array}$$

Notice that $\zeta_{il,k}^{\left(\epsilon \right)}$ and ${\tilde{y}}_{il,k}$ are mutually independent. Lemma 1 is given as follows to facilitate subsequent analysis.

**Lemma** **1** ([59])**.** *Supposing that the signal ${\bar{y}}_{il,k}$ is transmitted via a memoryless BSC with a crossover probability $\bar{\mu }$, and the expectation and the variance of the attained signal ${\tilde{y}}_{il,k}$ are shown below:*

(20) $$\begin{array}{c} E\left\{{\tilde{y}}_{il,k}\right\}=\left(1-2\bar{\mu }\right){\bar{y}}_{il,k},\;V\left\{{\tilde{y}}_{il,k}\right\}=L^{2}\phi \end{array}$$

*where $\phi ≜\frac{4\bar{\mu }\left(1-\bar{\mu }\right)\left(2^{2S}-1\right)}{3{\left(2^{S}-1\right)}^{2}}$ and the expectation is gotten concerning random variables $\mu_{il,k}^{\left(\epsilon \right)}$.*

Let

$$\begin{array}{cc} {Ϝ}_{i,k}≜ & {\left[\begin{array}{cccc} {Ϝ}_{i1,k} & {Ϝ}_{i2,k} & \cdots \; & {Ϝ}_{io_{y},k} \end{array}\right]}^{T}\left(Ϝ=y,\bar{y},\tilde{y},n\right). \end{array}$$

Combining (14) and (20), we acquire that

(21) $$\begin{array}{cc} E\{n_{i,k}\}= & 0,V\left\{n_{i,k}\right\}=\mathrm{diag}\left\{{\overset{ˇ}{\delta }}_{i1,k},{\overset{ˇ}{\delta }}_{i2,k},\ldots ,{\overset{ˇ}{\delta }}_{io_{y},k}\right\}\leq \left(1/4\right)\theta^{2}I_{\left(o_{y}\right)}, \end{array}$$

(22) $$\begin{array}{cc} E\{{\tilde{y}}_{i,k}\}= & \left(1-2\bar{\mu }\right){\bar{y}}_{i,k},V\left\{{\tilde{y}}_{i,k}\right\}=L^{2}\phi I_{\left(o_{y}\right)}. \end{array}$$

We recognize from (22) that the attained signals ${\tilde{y}}_{i,k}$ deviate from the quantized signals ${\bar{y}}_{i,k}$ with some distortions. For obviating such distortions, we signify

(23) $$\begin{array}{c} {\overset{˘}{y}}_{i,k}≜\frac{1}{1-2\bar{\mu }}{\tilde{y}}_{i,k} \end{array}$$

as the recovered measurement. According to (22), we obtain $E\left\{{\overset{˘}{y}}_{i,k}\right\}={\bar{y}}_{i,k}$. Define

(24) $$\begin{array}{c} m_{i,k}≜{\overset{˘}{y}}_{i,k}-{\bar{y}}_{i,k} \end{array}$$

as the stochastic noise showing impact from random bit errors. In consideration of (22)–(24), one yields

(25) $$\begin{array}{c} E\left\{m_{i,k}\right\}=0,V\left\{m_{i,k}\right\}=\left(1/{\left(1-2\bar{\mu }\right)}^{2}\right)L^{2}\phi I_{\left(o_{y}\right)}. \end{array}$$

Integrating (12) and (24), the recovered measurement is formulated below:

(26) $$\begin{array}{c} {\overset{˘}{y}}_{i,k}=y_{i,k}+n_{i,k}+m_{i,k}. \end{array}$$

#### 2.2.2. Deception Attacks

When signal is transmitted over the network, deception attacks may take place unavoidably. Assuming that the deception attack signal $d_{y_{i,k}}\in R^{o_{y}}$ satisfying Assumption 1 can replace the normal transmission signal completely in this paper. To manifest the occurrence of RODAs, a Bernoulli-distributed random variable $\alpha_{k}$ is adopted that satisfies $E\left\{\alpha_{k}\right\}=\bar{\alpha }$ and $V\left\{\alpha_{k}\right\}=\bar{\alpha }-{\bar{\alpha }}^{2}$. The transmitted signal suffering from deception attacks is described as

(27) $$\begin{array}{c} {\overset{´}{y}}_{i,k}=\left(1-\alpha_{k}\right)d_{y_{i,k}}+\alpha_{k}{\overset{˘}{y}}_{i,k}. \end{array}$$

**Assumption** **1** ([25])**.** *For a given real matrix F, the deception attack signal $d_{y_{i,k}}$ fulfils*

(28) $$\begin{array}{c} ∥d_{y_{i,k}}∥\;\leq ∥Fy_{i,k}∥. \end{array}$$

### 2.3. The Estimator Model

Based on the received measurement signal (27), the following estimator model is built:

(29) $$\begin{array}{cc} {\hat{x}}_{i,k+1}= & {\overset{˘}{A}}_{i}{\hat{x}}_{i,k}+{\overset{˘}{H}}_{i}h_{\overset{˘}{I}{\hat{x}}_{i,k}}+\sum_{j=1}^{N}p_{ij,q_{k}}{\overset{˘}{\Gamma }}_{q_{k}}{\hat{x}}_{j,k}+K_{i,q_{k}}\left({\overset{´}{y}}_{i,k}-\bar{\alpha }{\overset{˘}{C}}_{i}{\hat{x}}_{i,k}\right),\;i=1,2,\cdots ,N \end{array}$$

where ${\hat{x}}_{i,k}\in R^{o_{x}}$ means the estimate of ${\overset{˘}{x}}_{i,k}$. $K_{i,q_{k}}$$\left(i=1,2,\ldots ,N,q_{k}=1,2,\ldots ,U\right)$ is the estimator gain to be designed.

For symbol brevity, setting $♭≜q_{k}$. Letting $e_{i,k}≜{\overset{˘}{x}}_{i,k}-{\hat{x}}_{i,k}$ describe the estimation error of state and fault, whose expression is acquired below:

(30) $$\begin{array}{cc} e_{i,k+1}= & \left({\overset{˘}{A}}_{i}-\bar{\alpha }K_{i,♭}{\overset{˘}{C}}_{i}\right)e_{i,k}+\sum_{j=1}^{N}p_{ij,♭}{\overset{˘}{\Gamma }}_{♭}e_{j,k}-{\tilde{\alpha }}_{k}K_{i,♭}{\overset{˘}{C}}_{i}{\overset{˘}{x}}_{i,k}+\Delta {\overset{˘}{A}}_{i}{\overset{˘}{x}}_{i,k}+\Delta {\overset{˘}{H}}_{i}h_{\overset{˘}{I}{\overset{˘}{x}}_{i,k}}\\ \; & +{\overset{˘}{H}}_{i}h_{\overset{˘}{I}{\overset{˘}{x}}_{i,k}}-{\overset{˘}{H}}_{i}h_{\overset{˘}{I}{\hat{x}}_{i,k}}+{\overset{˘}{E}}_{i}w_{k}-\left(1-\bar{\alpha }\right)K_{i,♭}d_{y_{i,k}}+{\tilde{\alpha }}_{k}K_{i,♭}d_{y_{i,k}}\\ \; & -\bar{\alpha }K_{i,♭}n_{i,k}-{\tilde{\alpha }}_{k}K_{i,♭}n_{i,k}-\bar{\alpha }K_{i,♭}m_{i,k}-{\tilde{\alpha }}_{k}K_{i,♭}m_{i,k} \end{array}$$

where ${\tilde{\alpha }}_{k}≜\alpha_{k}-\bar{\alpha }$.

Defining

$$\begin{array}{cc} \; & {\overset{˘}{x}}_{k}≜{\left[\begin{array}{cccc} {\overset{˘}{x}}_{1,k}^{T} & {\overset{˘}{x}}_{2,k}^{T} & \cdots & {\overset{˘}{x}}_{N,k}^{T} \end{array}\right]}^{T},e_{k}≜{\left[\begin{array}{cccc} e_{1,k}^{T} & e_{2,k}^{T} & \cdots & e_{N,k}^{T} \end{array}\right]}^{T},\\ \; & n_{k}≜{\left[\begin{array}{cccc} n_{1,k}^{T} & n_{2,k}^{T} & \cdots & n_{N,k}^{T} \end{array}\right]}^{T},m_{k}≜{\left[\begin{array}{cccc} m_{1,k}^{T} & m_{2,k}^{T} & \cdots & m_{N,k}^{T} \end{array}\right]}^{T},\\ \; & {\bar{h}}_{{\overset{˘}{x}}_{k}}≜{\left[\begin{array}{cccc} h_{\overset{˘}{I}{\overset{˘}{x}}_{1,k}}^{T} & h_{\overset{˘}{I}{\overset{˘}{x}}_{2,k}}^{T} & \cdots & h_{\overset{˘}{I}{\overset{˘}{x}}_{N,k}}^{T} \end{array}\right]}^{T},{\bar{d}}_{y_{k}}≜{\left[\begin{array}{cccc} d_{y_{1,k}}^{T} & d_{y_{2,k}}^{T} & \cdots & d_{y_{N,k}}^{T} \end{array}\right]}^{T},\\ \; & {\hat{h}}_{e_{k}}≜{\left[\begin{array}{cccc} h_{\overset{˘}{I}{\overset{˘}{x}}_{1,k}}^{T}-h_{\overset{˘}{I}{\hat{x}}_{1,k}}^{T}\; & h_{\overset{˘}{I}{\overset{˘}{x}}_{2,k}}^{T}-h_{\overset{˘}{I}{\hat{x}}_{2,k}}^{T} & \cdots & h_{\overset{˘}{I}{\overset{˘}{x}}_{N,k}}^{T}-h_{\overset{˘}{I}{\hat{x}}_{N,k}}^{T} \end{array}\right]}^{T},\\ \; & \overset{˘}{A}≜\mathrm{diag}\left\{{\overset{˘}{A}}_{1},{\overset{˘}{A}}_{2},\ldots ,{\overset{˘}{A}}_{N}\right\},\Delta \overset{˘}{A}≜\mathrm{diag}\left\{\Delta {\overset{˘}{A}}_{1},\Delta {\overset{˘}{A}}_{2},\ldots ,\Delta {\overset{˘}{A}}_{N}\right\},\\ \; & \overset{˘}{C}≜\mathrm{diag}\left\{{\overset{˘}{C}}_{1},{\overset{˘}{C}}_{2},\ldots ,{\overset{˘}{C}}_{N}\right\},\overset{˘}{H}≜\mathrm{diag}\left\{{\overset{˘}{H}}_{1},{\overset{˘}{H}}_{2},\ldots ,{\overset{˘}{H}}_{N}\right\},\\ \; & \Delta \overset{˘}{H}≜\mathrm{diag}\left\{\Delta {\overset{˘}{H}}_{1},\Delta {\overset{˘}{H}}_{2},\ldots ,\Delta {\overset{˘}{H}}_{N}\right\},\overset{˘}{E}≜{\left[\begin{array}{cccc} {\overset{˘}{E}}_{1}^{T} & {\overset{˘}{E}}_{2}^{T} & \cdots & {\overset{˘}{E}}_{N}^{T} \end{array}\right]}^{T},\\ \; & K_{♭}≜\mathrm{diag}\left\{K_{1,♭},K_{2,♭},\ldots ,K_{N,♭}\right\}, \end{array}$$

the compact forms of (8) and (30) are derived below:

(31) $$\begin{array}{cc} {\overset{˘}{x}}_{k+1}= & \overset{˘}{A}{\overset{˘}{x}}_{k}+\Delta \overset{˘}{A}{\overset{˘}{x}}_{k}+\overset{˘}{H}{\bar{h}}_{{\overset{˘}{x}}_{k}}+\Delta \overset{˘}{H}{\bar{h}}_{{\overset{˘}{x}}_{k}}+\overset{˘}{E}w_{k}+\left(P_{♭}⊗{\overset{˘}{\Gamma }}_{♭}\right){\overset{˘}{x}}_{k} \end{array}$$

and

(32) $$\begin{array}{cc} e_{k+1}= & \left(\overset{˘}{A}-\bar{\alpha }K_{♭}\overset{˘}{C}+P_{♭}⊗{\overset{˘}{\Gamma }}_{♭}\right)e_{k}-{\tilde{\alpha }}_{k}K_{♭}\overset{˘}{C}{\overset{˘}{x}}_{k}+\overset{˘}{H}{\hat{h}}_{e_{k}}+\Delta \overset{˘}{A}{\overset{˘}{x}}_{k}+\Delta \overset{˘}{H}{\bar{h}}_{{\overset{˘}{x}}_{k}}+\overset{˘}{E}w_{k}\\ \; & -\left(1-\bar{\alpha }\right)K_{♭}{\bar{d}}_{y_{k}}+{\tilde{\alpha }}_{k}K_{♭}{\bar{d}}_{y_{k}}-\bar{\alpha }K_{♭}n_{k}-{\tilde{\alpha }}_{k}K_{♭}n_{k}-\bar{\alpha }K_{♭}m_{k}-{\tilde{\alpha }}_{k}K_{♭}m_{k}. \end{array}$$

Let $\eta_{k}≜{\left[\begin{array}{cc} {\overset{˘}{x}}_{k}^{T} & e_{k}^{T} \end{array}\right]}^{T}$ and ${\overset{˘}{h}}_{\eta_{k}}≜{\left[\begin{array}{cc} {\bar{h}}_{{\overset{˘}{x}}_{k}}^{T} & {\hat{h}}_{e_{k}}^{T} \end{array}\right]}^{T}$. The augmented EED is described from (31) and (32) as follows:

(33) $$\begin{array}{cc} \eta_{k+1}=\; & \left(A_{♭}-\bar{\alpha }K_{♭}C_{1}+\Delta A\right)\eta_{k}-{\tilde{\alpha }}_{k}K_{♭}C_{2}\eta_{k}+\left(H+\Delta H\right){\overset{˘}{h}}_{\eta_{k}}+Ew_{k}-\left(1-\bar{\alpha }\right)K_{♭}{\bar{d}}_{y_{k}}\\ \; & +{\tilde{\alpha }}_{k}K_{♭}{\bar{d}}_{y_{k}}-\bar{\alpha }K_{♭}n_{k}-{\tilde{\alpha }}_{k}K_{♭}n_{k}-\bar{\alpha }K_{♭}m_{k}-{\tilde{\alpha }}_{k}K_{♭}m_{k} \end{array}$$

where

$$\begin{array}{cc} \; & A_{♭}≜\mathrm{diag}\left\{\overset{˘}{A}+P_{♭}⊗{\overset{˘}{\Gamma }}_{♭},\overset{˘}{A}+P_{♭}⊗{\overset{˘}{\Gamma }}_{♭}\right\},C_{1}≜\left[\begin{array}{cc} 0 & \overset{˘}{C} \end{array}\right],H≜\mathrm{diag}\left\{\overset{˘}{H},\overset{˘}{H}\right\},\\ \; & \Delta A≜\left[\begin{array}{cc} \Delta \overset{˘}{A} & 0\\ \Delta \overset{˘}{A} & 0 \end{array}\right],\Delta H≜\left[\begin{array}{cc} \Delta \overset{˘}{H} & 0\\ \Delta \overset{˘}{H} & 0 \end{array}\right],E≜\left[\begin{array}{c} \overset{˘}{E}\\ \overset{˘}{E} \end{array}\right],K_{♭}≜\left[\begin{array}{c} 0\\ K_{♭} \end{array}\right],C_{2}≜\left[\begin{array}{cc} \overset{˘}{C} & 0 \end{array}\right]. \end{array}$$

**Definition** **1** ([5])**.** *The system (33) is said to be exponentially ultimately bounded in mean square if there exist scalars c>0, ς∈[0,1) and b≥0 such that the inequality below*

(34) $$\begin{array}{c} E\{∥\eta_{k}{∥}^{2}\}\;\leq c\varsigma^{k}{∥\eta_{0}∥}^{2}+b,\;k\geq 0 \end{array}$$

*holds for any solution $\eta_{k}$ with the initial condition $\eta_{0}$, where b denotes an ultimate upper bound in mean square of (33).*

**Figure 1:**
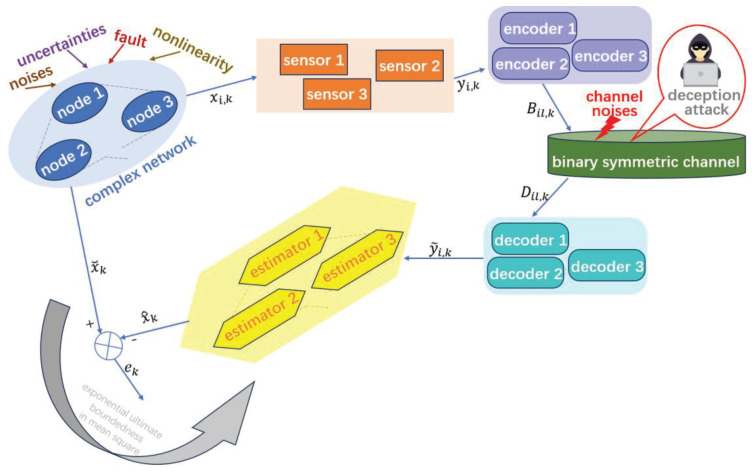
Illustration of the estimation issue for CN (1) using the case N=3.

The objective of this paper is to determine the gain matrix $K_{i,q_{k}}$(i=1,2,…,N,$q_{k}=1,2,\ldots ,U)$ in estimator (29) while the EED (33) is exponentially ultimately bounded in mean square. Figure 1 depicts the research structure of estimation issue in this paper.

## 3. Main Results

**Lemma** **2** ([60])**.** *Given a scalar ℘>0, real vectors $M\in R^{n}$ and $N\in R^{n}$, and a matrix $Q\in R^{n\times n}>0$, the matrix inequality below:*

(35) $$\begin{array}{c} M^{T}QN+N^{T}QM\leq ℘M^{T}QM+{℘}^{-1}N^{T}QN \end{array}$$

*holds.*

**Lemma** **3** ([6])**.** *Given constants 0≤γ≤1 and d. If {V(k)|k=0,1,2,…} is a nonnegative sequence satisfying*

$$\begin{array}{c} V(k+1)\leq \gamma V(k)+d, \end{array}$$

*then we have*

$$\begin{array}{c} V\left(k\right)\leq \left\{\begin{array}{cc} V(0)+kd & for\;\gamma =1,\\ \gamma^{k}V\left(0\right)+\frac{d(1-\gamma^{k})}{1-\gamma } & for\;\gamma \neq 1. \end{array}\right. \end{array}$$

**Theorem** **1.** *Given scalars 0<σ<1 and τ>0, and gain matrix $K_{♭}$. The state and fault estimator (29) is an exponentially ultimately bounded one in mean square for nonlinear CN (1) using BESs with switching couplings and deception attacks, if there exists a matrix $R_{♭}$ and scalars $\varrho_{1}>0$ and $\varrho_{2}>0$ satisfying the matrix inequality as follows:*

(36) $$\begin{array}{c} \Omega_{♭}≜\left[\begin{array}{ccc} \Omega_{11,♭} & \Omega_{12,♭} & \Omega_{13,♭}\\ {}* & \Omega_{22,♭} & \Omega_{23,♭}\\ {}* & * & \Omega_{33,♭} \end{array}\right]<0 \end{array}$$

*where*

$$\begin{array}{cc} \; & \Omega_{11,♭}≜{\left(A_{♭}-\bar{\alpha }K_{♭}C_{1}+\Delta A\right)}^{T}{\bar{R}}_{♭}\left(A_{♭}-\bar{\alpha }K_{♭}C_{1}+\Delta A\right)+{\tilde{\alpha }}^{2}C_{2}^{T}K_{♭}^{T}{\bar{R}}_{♭}K_{♭}C\\ \; & \;\;\;-\left(1-\sigma \right)R_{♭}-\varrho_{1}\left(I_{\left(2N\right)}⊗\bar{\Psi }\right)+\varrho_{2}I_{c}^{T}C_{F}I_{c},\\ \; & \Omega_{12,♭}≜{\left(A_{♭}-\bar{\alpha }K_{♭}C_{1}+\Delta A\right)}^{T}{\bar{R}}_{♭}\left(H+\Delta H\right)+\varrho_{1}{\left(I_{\left(2N\right)}⊗\tilde{\Psi }\right)}^{T},\\ \; & \Omega_{13,♭}≜-\left(1-\bar{\alpha }\right){\left(A_{♭}-\bar{\alpha }K_{♭}C_{1}+\Delta A\right)}^{T}{\bar{R}}_{♭}K_{♭}-{\tilde{\alpha }}^{2}C_{2}^{T}K_{♭}^{T}{\bar{R}}_{♭}K_{♭},\\ \; & \Omega_{22,♭}≜{\left(H+\Delta H\right)}^{T}{\bar{R}}_{♭}\left(H+\Delta H\right)-\varrho_{1}I_{\left(2No_{x}\right)},\\ \; & \Omega_{23,♭}≜-\left(1-\bar{\alpha }\right){\left(H+\Delta H\right)}^{T}{\bar{R}}_{♭}K_{♭},\\ \; & \Omega_{33,♭}≜{\left(1-\bar{\alpha }\right)}^{2}K_{♭}^{T}{\bar{R}}_{♭}K_{♭}+{\tilde{\alpha }}^{2}K_{♭}^{T}{\bar{R}}_{♭}K_{♭}-\varrho_{2}I_{\left(No_{y}\right)},\\ \; & {\bar{R}}_{♭}≜\sum_{℘=1}^{U}\pi_{♭℘}R_{℘},I_{c}≜\left[\begin{array}{cc} I_{\left(N\left(o_{x}+2o_{f}\right)\right)} & 0 \end{array}\right],\tilde{\alpha }≜\sqrt{\bar{\alpha }\left(1-\bar{\alpha }\right)},\\ \; & \bar{\Psi }≜\frac{{\overset{˘}{I}}^{T}\left(\Psi_{1}^{T}\Psi_{2}+\Psi_{2}^{T}\Psi_{1}\right)\overset{˘}{I}}{2},\tilde{\Psi }≜\frac{\left(\Psi_{1}+\Psi_{2}\right)\overset{˘}{I}}{2},\\ \; & C_{F}≜\mathrm{diag}\left\{{\overset{˘}{C}}_{1}^{T}F^{T}F{\overset{˘}{C}}_{1},{\overset{˘}{C}}_{2}^{T}F^{T}F{\overset{˘}{C}}_{2},\ldots ,{\overset{˘}{C}}_{N}^{T}F^{T}F{\overset{˘}{C}}_{N}\right\}. \end{array}$$

**Proof of Theorem** **1.** Construct Lyapunov function as follows:

(37) $$\begin{array}{c} V_{k,\eta_{k},q_{k}}≜\eta_{k}^{T}R_{q_{k}}\eta_{k}. \end{array}$$

Computing the expectation of the difference of $V_{k}$ along the trajectory of (33) with the expectation in (21) and (25), one has

(38) $$\begin{array}{cc} \; & E\{\Delta V_{k}\}\\ = & E\left\{V_{k+1,\eta_{k+1},q_{k+1}}|\eta_{k},q_{k}=♭\right\}-V_{k,\eta_{k},♭}\\ = & E\left\{\eta_{k+1}^{T}{\bar{R}}_{♭}\eta_{k+1}\right\}-\eta_{k}^{T}R_{♭}\eta_{k}\\ = & E\{\eta_{k}^{T}{\left(A_{♭}-\bar{\alpha }K_{♭}C_{1}+\Delta A\right)}^{T}{\bar{R}}_{♭}\left(A_{♭}-\bar{\alpha }K_{♭}C_{1}+\Delta A\right)\eta_{k}\\ \; & \;+{\overset{˘}{h}}_{\eta_{k}}^{T}{\left(H+\Delta H\right)}^{T}{\bar{R}}_{♭}\left(H+\Delta H\right){\overset{˘}{h}}_{\eta_{k}}+{\left(1-\bar{\alpha }\right)}^{2}{\bar{d}}_{y_{k}}^{T}K_{♭}^{T}{\bar{R}}_{♭}K_{♭}{\bar{d}}_{y_{k}}\\ \; & \;+2\eta_{k}^{T}{\left(A_{♭}-\bar{\alpha }K_{♭}C_{1}+\Delta A\right)}^{T}{\bar{R}}_{♭}\left(H+\Delta H\right){\overset{˘}{h}}_{\eta_{k}}\\ \; & \;-2\left(1-\bar{\alpha }\right)\eta_{k}^{T}{\left(A_{♭}-\bar{\alpha }K_{♭}C_{1}+\Delta A\right)}^{T}{\bar{R}}_{♭}K_{♭}{\bar{d}}_{y_{k}}\\ \; & \;-2\left(1-\bar{\alpha }\right){\overset{˘}{h}}_{\eta_{k}}^{T}{\left(H+\Delta H\right)}^{T}{\bar{R}}_{♭}K_{♭}{\bar{d}}_{y_{k}}+{\tilde{\alpha }}^{2}\eta_{k}^{T}C_{2}^{T}K_{♭}^{T}{\bar{R}}_{♭}K_{♭}C_{2}\eta_{k}\\ \; & \;+{\tilde{\alpha }}^{2}{\bar{d}}_{y_{k}}^{T}K_{♭}^{T}{\bar{R}}_{♭}K_{♭}{\bar{d}}_{y_{k}}-2{\tilde{\alpha }}^{2}\eta_{k}^{T}C_{2}^{T}K_{♭}^{T}{\bar{R}}_{♭}K_{♭}{\bar{d}}_{y_{k}}+w_{k}^{T}E^{T}{\bar{R}}_{♭}Ew_{k}\\ \; & \;+{\bar{\alpha }}^{2}n_{k}^{T}K_{♭}^{T}{\bar{R}}_{♭}K_{♭}n_{k}+{\bar{\alpha }}^{2}m_{k}^{T}K_{♭}^{T}{\bar{R}}_{♭}K_{♭}m_{k}+2{\bar{\alpha }}^{2}n_{k}^{T}K_{♭}^{T}{\bar{R}}_{♭}K_{♭}m_{k}\\ \; & \;+{\tilde{\alpha }}^{2}n_{k}^{T}K_{♭}^{T}{\bar{R}}_{♭}K_{♭}n_{k}+{\tilde{\alpha }}^{2}m_{k}^{T}K_{♭}^{T}{\bar{R}}_{♭}K_{♭}m_{k}+2{\tilde{\alpha }}^{2}n_{k}^{T}K_{♭}^{T}{\bar{R}}_{♭}K_{♭}m_{k}-\eta_{k}^{T}R_{♭}\eta_{k}\}\\ = & E\{\eta_{k}^{T}{\left(A_{♭}-\bar{\alpha }K_{♭}C_{1}+\Delta A\right)}^{T}{\bar{R}}_{♭}\left(A_{♭}-\bar{\alpha }K_{♭}C_{1}+\Delta A\right)\eta_{k}\\ \; & \;+{\overset{˘}{h}}_{\eta_{k}}^{T}{\left(H+\Delta H\right)}^{T}{\bar{R}}_{♭}\left(H+\Delta H\right){\overset{˘}{h}}_{\eta_{k}}+{\left(1-\bar{\alpha }\right)}^{2}{\bar{d}}_{y_{k}}^{T}K_{♭}^{T}{\bar{R}}_{♭}K_{♭}{\bar{d}}_{y_{k}}\\ \; & \;+2\eta_{k}^{T}{\left(A_{♭}-\bar{\alpha }K_{♭}C_{1}+\Delta A\right)}^{T}{\bar{R}}_{♭}\left(H+\Delta H\right){\overset{˘}{h}}_{\eta_{k}}\\ \; & \;-2\left(1-\bar{\alpha }\right)\eta_{k}^{T}{\left(A_{♭}-\bar{\alpha }K_{♭}C_{1}+\Delta A\right)}^{T}{\bar{R}}_{♭}K_{♭}{\bar{d}}_{y_{k}}\\ \; & \;-2\left(1-\bar{\alpha }\right){\overset{˘}{h}}_{\eta_{k}}^{T}{\left(H+\Delta H\right)}^{T}{\bar{R}}_{♭}K_{♭}{\bar{d}}_{y_{k}}+{\tilde{\alpha }}^{2}\eta_{k}^{T}C_{2}^{T}K_{♭}^{T}{\bar{R}}_{♭}K_{♭}C_{2}\eta_{k}\\ \; & \;+{\tilde{\alpha }}^{2}{\bar{d}}_{y_{k}}^{T}K_{♭}^{T}{\bar{R}}_{♭}K_{♭}{\bar{d}}_{y_{k}}-2{\tilde{\alpha }}^{2}\eta_{k}^{T}C_{2}^{T}K_{♭}^{T}{\bar{R}}_{♭}K_{♭}{\bar{d}}_{y_{k}}+w_{k}^{T}E^{T}{\bar{R}}_{♭}Ew_{k}\\ \; & \;+\bar{\alpha }n_{k}^{T}K_{♭}^{T}{\bar{R}}_{♭}K_{♭}n_{k}+\bar{\alpha }m_{k}^{T}K_{♭}^{T}{\bar{R}}_{♭}K_{♭}m_{k}+2\bar{\alpha }n_{k}^{T}K_{♭}^{T}{\bar{R}}_{♭}K_{♭}m_{k}-\eta_{k}^{T}R_{♭}\eta_{k}\}. \end{array}$$

In terms of (3), we have

$$\begin{array}{cc} \; & {\left[h_{x_{i,k}}-\Psi_{1}x_{i,k}\right]}^{T}\left[h_{x_{i,k}}-\Psi_{2}x_{i,k}\right]\leq 0,i=1,2,\ldots ,N, \end{array}$$

i.e.,

(39) $$\begin{array}{cc} \; & {\left[h_{\overset{˘}{I}{\overset{˘}{x}}_{i,k}}-\Psi_{1}\overset{˘}{I}{\overset{˘}{x}}_{i,k}\right]}^{T}\left[h_{\overset{˘}{I}{\overset{˘}{x}}_{i,k}}-\Psi_{2}\overset{˘}{I}{\overset{˘}{x}}_{i,k}\right]\leq 0, \end{array}$$

(40) $$\begin{array}{cc} \; & {\left[h_{\overset{˘}{I}{\overset{˘}{x}}_{i,k}}-h_{\overset{˘}{I}{\hat{x}}_{i,k}}-\Psi_{1}\left(\overset{˘}{I}{\overset{˘}{x}}_{i,k}-\overset{˘}{I}{\hat{x}}_{i,k}\right)\right]}^{T}\left[h_{\overset{˘}{I}{\overset{˘}{x}}_{i,k}}-h_{\overset{˘}{I}{\hat{x}}_{i,k}}-\Psi_{2}\left(\overset{˘}{I}{\overset{˘}{x}}_{i,k}-\overset{˘}{I}{\hat{x}}_{i,k}\right)\right]\leq 0. \end{array}$$

Rewrite (39) and (40) as

(41) $$\begin{array}{cc} \; & {\left[\begin{array}{c} {\overset{˘}{x}}_{i,k}\\ h_{\overset{˘}{I}{\overset{˘}{x}}_{i,k}} \end{array}\right]}^{T}\left[\begin{array}{cc} \bar{\Psi } & *\\ -\tilde{\Psi } & I_{\left(o_{x}\right)} \end{array}\right]\left[\begin{array}{c} {\overset{˘}{x}}_{i,k}\\ h_{\overset{˘}{I}{\overset{˘}{x}}_{i,k}} \end{array}\right]\leq 0, \end{array}$$

(42) $$\begin{array}{cc} \; & {\left[\begin{array}{c} {\overset{˘}{x}}_{i,k}-{\hat{x}}_{i,k}\\ h_{\overset{˘}{I}{\overset{˘}{x}}_{i,k}}-h_{\overset{˘}{I}{\hat{x}}_{i,k}} \end{array}\right]}^{T}\left[\begin{array}{cc} \bar{\Psi } & *\\ -\tilde{\Psi } & I_{\left(o_{x}\right)} \end{array}\right]\left[\begin{array}{c} {\overset{˘}{x}}_{i,k}-{\hat{x}}_{i,k}\\ h_{\overset{˘}{I}{\overset{˘}{x}}_{i,k}}-h_{\overset{˘}{I}{\hat{x}}_{i,k}} \end{array}\right]\leq 0,i=1,2,\ldots ,N. \end{array}$$

Furthermore, one obtains from (41) and (42) that

(43) $$\begin{array}{cc} \; & {\left[\begin{array}{c} {\overset{˘}{x}}_{k}\\ {\bar{h}}_{{\overset{˘}{x}}_{k}} \end{array}\right]}^{T}\left[\begin{array}{cc} I_{\left(N\right)}⊗\bar{\Psi } & *\\ -I_{\left(N\right)}⊗\tilde{\Psi } & I_{\left(No_{x}\right)} \end{array}\right]\left[\begin{array}{c} {\overset{˘}{x}}_{k}\\ {\bar{h}}_{{\overset{˘}{x}}_{k}} \end{array}\right]\leq 0, \end{array}$$

(44) $$\begin{array}{cc} \; & {\left[\begin{array}{c} e_{k}\\ {\hat{h}}_{e_{k}} \end{array}\right]}^{T}\left[\begin{array}{cc} I_{\left(N\right)}⊗\bar{\Psi } & *\\ -I_{\left(N\right)}⊗\tilde{\Psi } & I_{\left(No_{x}\right)} \end{array}\right]\left[\begin{array}{c} e_{k}\\ {\hat{h}}_{e_{k}} \end{array}\right]\leq 0. \end{array}$$

Based on (43) and (44), one yields

(45) $$\begin{array}{c} {\left[\begin{array}{c} \eta_{k}\\ {\overset{˘}{h}}_{\eta_{k}} \end{array}\right]}^{T}\left[\begin{array}{cc} I_{\left(2N\right)}⊗\bar{\Psi } & *\\ -I_{\left(2N\right)}⊗\tilde{\Psi } & I_{\left(2No_{x}\right)} \end{array}\right]\left[\begin{array}{c} \eta_{k}\\ {\overset{˘}{h}}_{\eta_{k}} \end{array}\right]\leq 0. \end{array}$$

Taking account of (28), one gets

(46) $$\begin{array}{c} {\left[\begin{array}{c} {\overset{˘}{x}}_{i,k}\\ d_{y_{i,k}} \end{array}\right]}^{T}\left[\begin{array}{cc} -{\overset{˘}{C}}_{i}^{T}F^{T}F{\overset{˘}{C}}_{i} & *\\ 0 & I_{\left(o_{y}\right)} \end{array}\right]\left[\begin{array}{c} {\overset{˘}{x}}_{i,k}\\ d_{y_{i,k}} \end{array}\right]\leq 0,i=1,2,\ldots ,N. \end{array}$$

Moreover, one acquires from (46) that

(47) $$\begin{array}{c} {\left[\begin{array}{c} {\overset{˘}{x}}_{k}\\ {\bar{d}}_{y_{k}} \end{array}\right]}^{T}\left[\begin{array}{cc} -C_{F} & *\\ 0 & I_{\left(No_{y}\right)} \end{array}\right]\left[\begin{array}{c} {\overset{˘}{x}}_{k}\\ {\bar{d}}_{y_{k}} \end{array}\right]\leq 0, \end{array}$$

i.e.,

(48) $$\begin{array}{c} {\left[\begin{array}{c} I_{c}\eta_{k}\\ {\bar{d}}_{y_{k}} \end{array}\right]}^{T}\left[\begin{array}{cc} -C_{F} & *\\ 0 & I_{\left(No_{y}\right)} \end{array}\right]\left[\begin{array}{c} I_{c}\eta_{k}\\ {\bar{d}}_{y_{k}} \end{array}\right]\leq 0. \end{array}$$

Note that (48) is equivalent to

(49) $$\begin{array}{c} {\left[\begin{array}{c} \eta_{k}\\ {\bar{d}}_{y_{k}} \end{array}\right]}^{T}\left[\begin{array}{cc} -I_{c}^{T}C_{F}I_{c} & *\\ 0 & I_{\left(No_{y}\right)} \end{array}\right]\left[\begin{array}{c} \eta_{k}\\ {\bar{d}}_{y_{k}} \end{array}\right]\leq 0. \end{array}$$

Using Lemma 2, we know that

(50) $$\begin{array}{cc} \; & \bar{\alpha }n_{k}^{T}K_{♭}^{T}{\bar{R}}_{♭}K_{♭}m_{k}+\bar{\alpha }m_{k}^{T}K_{♭}^{T}{\bar{R}}_{♭}K_{♭}n_{k}\leq \tau \bar{\alpha }n_{k}^{T}K_{♭}^{T}{\bar{R}}_{♭}K_{♭}n_{k}+\tau^{-1}\bar{\alpha }m_{k}^{T}K_{♭}^{T}{\bar{R}}_{♭}K_{♭}m_{k}. \end{array}$$

Combining (45), (49) and (50), the variance in (21) and (25), and the trace property of matrix multiplication, we get

(51) $$\begin{array}{cc} \; & E\{\Delta V_{k}\}\\ \leq & E\{\eta_{k}^{T}{\left(A_{♭}-\bar{\alpha }K_{♭}C_{1}+\Delta A\right)}^{T}{\bar{R}}_{♭}\left(A_{♭}-\bar{\alpha }K_{♭}C_{1}+\Delta A\right)\eta_{k}\\ \; & \;+{\overset{˘}{h}}_{\eta_{k}}^{T}{\left(H+\Delta H\right)}^{T}{\bar{R}}_{♭}\left(H+\Delta H\right){\overset{˘}{h}}_{\eta_{k}}+{\left(1-\bar{\alpha }\right)}^{2}{\bar{d}}_{y_{k}}^{T}K_{♭}^{T}{\bar{R}}_{♭}K_{♭}{\bar{d}}_{y_{k}}\\ \; & \;+2\eta_{k}^{T}{\left(A_{♭}-\bar{\alpha }K_{♭}C_{1}+\Delta A\right)}^{T}{\bar{R}}_{♭}\left(H+\Delta H\right){\overset{˘}{h}}_{\eta_{k}}\\ \; & \;-2\left(1-\bar{\alpha }\right)\eta_{k}^{T}{\left(A_{♭}-\bar{\alpha }K_{♭}C_{1}+\Delta A\right)}^{T}{\bar{R}}_{♭}K_{♭}{\bar{d}}_{y_{k}}\\ \; & \;-2\left(1-\bar{\alpha }\right){\overset{˘}{h}}_{\eta_{k}}^{T}{\left(H+\Delta H\right)}^{T}{\bar{R}}_{♭}K_{♭}{\bar{d}}_{y_{k}}+{\tilde{\alpha }}^{2}\eta_{k}^{T}C_{2}^{T}K_{♭}^{T}{\bar{R}}_{♭}K_{♭}C_{2}\eta_{k}\\ \; & \;+{\tilde{\alpha }}^{2}{\bar{d}}_{y_{k}}^{T}K_{♭}^{T}{\bar{R}}_{♭}K_{♭}{\bar{d}}_{y_{k}}-2{\tilde{\alpha }}^{2}\eta_{k}^{T}C_{2}^{T}K_{♭}^{T}{\bar{R}}_{♭}K_{♭}{\bar{d}}_{y_{k}}\\ \; & \;+\mathrm{tr}\left(w_{k}^{T}E^{T}{\bar{R}}_{♭}Ew_{k}\right)+\mathrm{tr}\left(\bar{\alpha }\left(1+\tau \right)n_{k}^{T}K_{♭}^{T}{\bar{R}}_{♭}K_{♭}n_{k}\right)\\ \; & \;+\mathrm{tr}\left(\bar{\alpha }\left(1+\tau^{-1}\right)m_{k}^{T}K_{♭}^{T}{\bar{R}}_{♭}K_{♭}m_{k}\right)-\eta_{k}^{T}R_{♭}\eta_{k}\}\\ \; & \;-\varrho_{1}(\eta_{k}^{T}\left(I_{\left(2N\right)}⊗\bar{\Psi }\right)\eta_{k}-\eta_{k}^{T}{\left(I_{\left(2N\right)}⊗\tilde{\Psi }\right)}^{T}{\overset{˘}{h}}_{\eta_{k}}\\ \; & \;-{\overset{˘}{h}}_{\eta_{k}}^{T}\left(I_{\left(2N\right)}⊗\tilde{\Psi }\right)\eta_{k}+{\overset{˘}{h}}_{\eta_{k}}^{T}{\overset{˘}{h}}_{\eta_{k}})-\varrho_{2}\left(-\eta_{k}^{T}I_{c}^{T}C_{F}I_{c}\eta_{k}+{\bar{d}}_{y_{k}}^{T}{\bar{d}}_{y_{k}}\right)\\ = & E\{\eta_{k}^{T}{\left(A_{♭}-\bar{\alpha }K_{♭}C_{1}+\Delta A\right)}^{T}{\bar{R}}_{♭}\left(A_{♭}-\bar{\alpha }K_{♭}C_{1}+\Delta A\right)\eta_{k}\\ \; & \;+{\overset{˘}{h}}_{\eta_{k}}^{T}{\left(H+\Delta H\right)}^{T}{\bar{R}}_{♭}\left(H+\Delta H\right){\overset{˘}{h}}_{\eta_{k}}+{\left(1-\bar{\alpha }\right)}^{2}{\bar{d}}_{y_{k}}^{T}K_{♭}^{T}{\bar{R}}_{♭}K_{♭}{\bar{d}}_{y_{k}}\\ \; & \;+2\eta_{k}^{T}{\left(A_{♭}-\bar{\alpha }K_{♭}C_{1}+\Delta A\right)}^{T}{\bar{R}}_{♭}\left(H+\Delta H\right){\overset{˘}{h}}_{\eta_{k}}\\ \; & \;-2\left(1-\bar{\alpha }\right)\eta_{k}^{T}{\left(A_{♭}-\bar{\alpha }K_{♭}C_{1}+\Delta A\right)}^{T}{\bar{R}}_{♭}K_{♭}{\bar{d}}_{y_{k}}\\ \; & \;-2\left(1-\bar{\alpha }\right){\overset{˘}{h}}_{\eta_{k}}^{T}{\left(H+\Delta H\right)}^{T}{\bar{R}}_{♭}K_{♭}{\bar{d}}_{y_{k}}+{\tilde{\alpha }}^{2}\eta_{k}^{T}C_{2}^{T}K_{♭}^{T}{\bar{R}}_{♭}K_{♭}C_{2}\eta_{k}\\ \; & \;+{\tilde{\alpha }}^{2}{\bar{d}}_{y_{k}}^{T}K_{♭}^{T}{\bar{R}}_{♭}K_{♭}{\bar{d}}_{y_{k}}-2{\tilde{\alpha }}^{2}\eta_{k}^{T}C_{2}^{T}K_{♭}^{T}{\bar{R}}_{♭}K_{♭}{\bar{d}}_{y_{k}}\\ \; & \;+\mathrm{tr}\left(E^{T}{\bar{R}}_{♭}Ew_{k}w_{k}^{T}\right)+\mathrm{tr}\left(\bar{\alpha }\left(1+\tau \right)K_{♭}^{T}{\bar{R}}_{♭}K_{♭}n_{k}n_{k}^{T}\right)\\ \; & \;+\mathrm{tr}\left(\bar{\alpha }\left(1+\tau^{-1}\right)K_{♭}^{T}{\bar{R}}_{♭}K_{♭}m_{k}m_{k}^{T}\right)-\eta_{k}^{T}R_{♭}\eta_{k}\}\\ \; & \;-\varrho_{1}(\eta_{k}^{T}\left(I_{\left(2N\right)}⊗\bar{\Psi }\right)\eta_{k}-\eta_{k}^{T}{\left(I_{\left(2N\right)}⊗\tilde{\Psi }\right)}^{T}{\overset{˘}{h}}_{\eta_{k}}\\ \; & \;-{\overset{˘}{h}}_{\eta_{k}}^{T}\left(I_{\left(2N\right)}⊗\tilde{\Psi }\right)\eta_{k}+{\overset{˘}{h}}_{\eta_{k}}^{T}{\overset{˘}{h}}_{\eta_{k}})-\varrho_{2}\left(-\eta_{k}^{T}I_{c}^{T}C_{F}I_{c}\eta_{k}+{\bar{d}}_{y_{k}}^{T}{\bar{d}}_{y_{k}}\right)\\ \; & \;+E\left\{\sigma \eta_{k}^{T}R_{♭}\eta_{k}\right\}-E\left\{\sigma \eta_{k}^{T}R_{♭}\eta_{k}\right\}\\ = & {\tilde{\eta }}_{k}^{T}\Omega_{♭}{\tilde{\eta }}_{k}-E\left\{\sigma V_{k,\eta_{k},q_{k}}\right\}+\mathrm{tr}\left(\Xi_{♭}\right) \end{array}$$

where

$$\begin{array}{cc} \; & {\tilde{\eta }}_{k}≜{\left[\begin{array}{ccc} \eta_{k}^{T} & {\overset{˘}{h}}_{\eta_{k}}^{T} & {\bar{d}}_{y_{k}}^{T} \end{array}\right]}^{T},\\ \; & \Xi_{♭}≜\mathrm{diag}\left\{w_{0}^{2}E^{T}{\bar{R}}_{♭}E,\left(\theta^{2}/4\right)\bar{\alpha }\left(1+\tau \right)K_{♭}^{T}{\bar{R}}_{♭}K_{♭},\left(L^{2}\phi /{\left(1-2\bar{\mu }\right)}^{2}\right)\bar{\alpha }\left(1+\tau^{-1}\right)K_{♭}^{T}{\bar{R}}_{♭}K_{♭}\right\}. \end{array}$$

By virtue of (36), we find from (51) that

(52) $$\begin{array}{c} E\left\{\Delta V_{k}\right\}<-\sigma E\left\{V_{k,\eta_{k},q_{k}}\right\}+\mathrm{tr}\left(\Xi_{♭}\right), \end{array}$$

i.e.,

(53) $$\begin{array}{c} E\left\{V_{k+1,\eta_{k+1},q_{k+1}}|\eta_{k},q_{k}=♭\right\}<\left(1-\sigma \right)E\left\{V_{k,\eta_{k},q_{k}}\right\}+\mathrm{tr}\left(\Xi_{♭}\right). \end{array}$$

According to Lemma 3 and (37), we notice from (53) that

(54) $$\begin{array}{cc} E\{V_{k,\eta_{k},q_{k}}\}< & {\left(1-\sigma \right)}^{k}V_{0}+\frac{\mathrm{tr}\left(\Xi_{♭}\right)\left(1-{\left(1-\sigma \right)}^{k}\right)}{\sigma }\\ \leq & {\left(1-\sigma \right)}^{k}\lambda_{\max}\left(R_{q_{0}}\right){∥\eta_{0}∥}^{2}+\frac{\mathrm{tr}(\Xi_{♭})}{\sigma }. \end{array}$$

Noting that $E\left\{V_{k,\eta_{k},q_{k}}\right\}\geq \lambda_{\min}\left(R_{♭}\right)E\{∥\eta_{k}{∥}^{2}\}$, the following inequality is obtained from (54):

(55) $$\begin{array}{c} E\{∥\eta_{k}{∥}^{2}\}\leq \frac{\lambda_{\max}\left(R_{q_{0}}\right)}{\lambda_{\min}\left(R_{♭}\right)}{\left(1-\sigma \right)}^{k}{∥\eta_{0}∥}^{2}+\frac{\mathrm{tr}(\Xi_{♭})}{\sigma \lambda_{\min}\left(R_{♭}\right)}. \end{array}$$

In terms of (34), it is indicated from (55) that the system (33) is exponentially ultimately bounded in mean square, and an ultimate upper bound in mean square is $\frac{\mathrm{tr}(\Xi_{♭})}{\sigma \lambda_{\min}\left(R_{♭}\right)}$. The proof is finished now. □

**Theorem** **2.** *Given scalars 0<σ<1 and τ>0. The state and fault estimator (29) is an exponentially ultimately bounded one in mean square for nonlinear CN (1) using BESs with uncertainties, switching couplings and deception attacks, if there exist matrices $R_{♭}≜\mathrm{diag}\left\{R_{1,♭},R_{2,♭}\right\}>0$ ($R_{1,♭}≜\mathrm{diag}\left\{R_{11,♭},R_{12,♭},\ldots ,R_{1N,♭}\right\}$ and $R_{2,♭}≜\mathrm{diag}\left\{R_{21,♭},R_{22,♭},\ldots ,R_{2N,♭}\right\}$, ♭=1,2,…,U), ${\bar{\Lambda }}_{♭}≜\left[\begin{array}{c} 0\\ \Lambda_{♭} \end{array}\right]$ ($\Lambda_{♭}≜\mathrm{diag}\left\{\Lambda_{1,♭},\Lambda_{2,♭},\ldots ,\Lambda_{N,♭}\right\}$) and $\Phi ≜\mathrm{diag}\{\Phi_{1},\Phi_{2},\Phi_{3}\}>0$, and scalars $\varrho_{1}>0$, $\varrho_{2}>0$ and ℏ>0 satisfying the following matrix inequalities:*

(56) $$\begin{array}{cc} \; & \left[\begin{array}{ccccccc} {\overset{˘}{\Omega }}_{11,♭} & * & * & * & * & * & *\\ {\overset{˘}{\Omega }}_{21} & {\overset{˘}{\Omega }}_{22} & * & * & * & * & *\\ 0 & 0 & {\overset{˘}{\Omega }}_{33} & * & * & * & *\\ {\overset{˘}{\Omega }}_{41,♭}\; & {\bar{R}}_{♭}H\; & {\overset{˘}{\Omega }}_{43,♭} & -{\bar{R}}_{♭} & * & * & *\\ -\tilde{\alpha }{\bar{\Lambda }}_{♭}C_{2}\; & 0\; & \tilde{\alpha }{\bar{\Lambda }}_{♭} & 0 & -{\bar{R}}_{♭} & * & *\\ 0 & 0 & 0 & {\hat{J}}^{T}{\bar{R}}_{♭}^{T} & 0 & -\hbar I & *\\ \hbar {\hat{J}}_{3A} & \hbar {\hat{J}}_{3H} & 0 & 0 & 0 & 0 & -\hbar I \end{array}\right]<0, \end{array}$$

(57) $$\begin{array}{cc} \; & \left[\begin{array}{cc} -\Phi & *\\ \Theta_{♭} & -I_{\left(3\right)}⊗{\bar{R}}_{♭} \end{array}\right]<0 \end{array}$$

*where*

$$\begin{array}{cc} \; & {\bar{R}}_{2i,♭}≜\sum_{℘=1}^{U}\pi_{♭℘}R_{2i,℘},\\ \; & {\overset{˘}{\Omega }}_{11,♭}≜-\left(1-\sigma \right)R_{♭}-\varrho_{1}\left(I_{\left(2N\right)}⊗\bar{\Psi }\right)+\varrho_{2}I_{c}^{T}C_{F}I_{c},\\ \; & {\overset{˘}{\Omega }}_{21}≜\varrho_{1}\left(I_{\left(2N\right)}⊗\tilde{\Psi }\right),\\ \; & {\overset{˘}{\Omega }}_{22}≜-\varrho_{1}I_{\left(2No_{x}\right)},\\ \; & {\overset{˘}{\Omega }}_{33}≜-\varrho_{2}I_{\left(No_{y}\right)},\\ \; & {\overset{˘}{\Omega }}_{41,♭}≜{\bar{R}}_{♭}A_{♭}-\bar{\alpha }{\bar{\Lambda }}_{♭}C_{1},\\ \; & {\overset{˘}{\Omega }}_{43,♭}≜-\left(1-\bar{\alpha }\right){\bar{\Lambda }}_{♭},\\ \; & \Theta_{♭}≜\mathrm{diag}\left\{w_{0}{\bar{R}}_{♭}E,\left(\theta \sqrt{\bar{\alpha }\left(1+\tau \right)}/2\right){\bar{\Lambda }}_{♭},\left(L\sqrt{\phi \bar{\alpha }\left(1+\tau^{-1}\right)}/\left(1-2\bar{\mu }\right)\right){\bar{\Lambda }}_{♭}\right\}. \end{array}$$

*Additionally, the minimum of the ultimate upper bound of $E\{∥\eta_{k}{∥}^{2}\}$ is obtained through tackling the optimization issue below:*

(58) $$\begin{array}{c} \min_{subject\;to(56)-(57)}\left\{\mathrm{tr}\left(\Phi \right)\right\}. \end{array}$$

*The estimator gain matrix in (29) is denoted as*

$$K_{i,♭}={\left({\bar{R}}_{2i,♭}\right)}^{-1}\Lambda_{i,♭}\;\;\left(i=1,2,\ldots ,N\right).$$

**Proof of Theorem** **2.** $\Omega_{♭}$ in (36) is equivalent to

(59) $$\begin{array}{cc} \Omega_{♭}= & {\overset{˘}{\Omega }}_{♭}+\Pi_{1,♭}^{T}{\bar{R}}_{♭}\Pi_{1,♭}+\Pi_{2,♭}^{T}{\bar{R}}_{♭}\Pi_{2,♭}\\ = & {\overset{˘}{\Omega }}_{♭}+\left[\begin{array}{cc} \Pi_{1,♭}^{T} & \Pi_{2,♭}^{T} \end{array}\right]\left(I_{\left(2\right)}⊗{\bar{R}}_{♭}\right){\left[\begin{array}{cc} \Pi_{1,♭}^{T} & \Pi_{2,♭}^{T} \end{array}\right]}^{T}\\ = & {\overset{˘}{\Omega }}_{♭}+\left[\begin{array}{cc} \Pi_{1,♭}^{T} & \Pi_{2,♭}^{T} \end{array}\right]\left(I_{\left(2\right)}⊗{\bar{R}}_{♭}\right){\left(I_{\left(2\right)}⊗{\bar{R}}_{♭}\right)}^{-1}\left(I_{\left(2\right)}⊗{\bar{R}}_{♭}\right){\left[\begin{array}{cc} \Pi_{1,♭}^{T} & \Pi_{2,♭}^{T} \end{array}\right]}^{T}\\ = & {\overset{˘}{\Omega }}_{♭}+\left[\begin{array}{cc} \Pi_{1,♭}^{T}{\bar{R}}_{♭} & \Pi_{2,♭}^{T}{\bar{R}}_{♭} \end{array}\right]{\left(I_{\left(2\right)}⊗{\bar{R}}_{♭}\right)}^{-1}{\left[\begin{array}{cc} {\left({\bar{R}}_{♭}\Pi_{1,♭}\right)}^{T} & {\left({\bar{R}}_{♭}\Pi_{2,♭}\right)}^{T} \end{array}\right]}^{T}\\ < & 0 \end{array}$$

where

$$\begin{array}{cc} \; & {\overset{˘}{\Omega }}_{♭}≜\left[\begin{array}{ccc} {\overset{˘}{\Omega }}_{11,♭}\; & \varrho_{1}{\left(I_{\left(2N\right)}⊗\tilde{\Psi }\right)}^{T}\; & 0\\ {}* & -\varrho_{1}I_{\left(2No_{x}\right)} & 0\\ {}* & * & -\varrho_{2}I_{\left(No_{y}\right)} \end{array}\right],\\ \; & \Pi_{1,♭}≜\left[\begin{array}{ccc} A_{♭}-\bar{\alpha }K_{♭}C_{1}+\Delta A\; & H+\Delta H\; & -\left(1-\bar{\alpha }\right)K_{♭} \end{array}\right],\Pi_{2,♭}≜\left[\begin{array}{ccc} -\tilde{\alpha }K_{♭}C_{2}\; & 0\; & \tilde{\alpha }K_{♭} \end{array}\right]. \end{array}$$

On the basis of Schur Complement Lemma, (59) holds as long as the following inequality holds:

(60) $$\begin{array}{c} \left[\begin{array}{ccc} {\overset{˘}{\Omega }}_{♭} & * & *\\ {\bar{R}}_{♭}\Pi_{1,♭} & -{\bar{R}}_{♭} & *\\ {\bar{R}}_{♭}\Pi_{2,♭} & 0 & -{\bar{R}}_{♭} \end{array}\right]<0, \end{array}$$

i.e.,

(61) $$\begin{array}{cc} \; & \left[\begin{array}{ccccc} {\overset{˘}{\Omega }}_{11,♭} & * & * & * & *\\ \varrho_{1}\left(I_{\left(2N\right)}⊗\tilde{\Psi }\right) & -\varrho_{1}I_{\left(2No_{x}\right)}\; & * & * & *\\ 0 & 0 & -\varrho_{2}I_{\left(No_{y}\right)} & * & *\\ {\hat{\Omega }}_{41,♭}\; & {\bar{R}}_{♭}H+{\bar{R}}_{♭}\Delta H\; & -\left(1-\bar{\alpha }\right){\bar{R}}_{♭}K_{♭} & -{\bar{R}}_{♭} & *\\ -\tilde{\alpha }{\bar{R}}_{♭}K_{♭}C_{2}\; & 0\; & \tilde{\alpha }{\bar{R}}_{♭}K_{♭}\; & 0 & -{\bar{R}}_{♭} \end{array}\right]<0 \end{array}$$

where ${\hat{\Omega }}_{41,♭}≜{\bar{R}}_{♭}A_{♭}-\bar{\alpha }{\bar{R}}_{♭}K_{♭}C_{1}+{\bar{R}}_{♭}\Delta A$.

Split (61) as follows:

(62) $$\begin{array}{cc} \; & {ℶ}_{1,♭}+{ℶ}_{2,♭}+{ℶ}_{2,♭}^{T}<0 \end{array}$$

where

$$\begin{array}{cc} {ℶ}_{1,♭}≜ & \left[\begin{array}{ccccc} {\overset{˘}{\Omega }}_{11,♭} & * & * & * & *\\ \varrho_{1}\left(I_{\left(2N\right)}⊗\tilde{\Psi }\right) & -\varrho_{1}I_{\left(2No_{x}\right)}\; & * & * & *\\ 0 & 0 & -\varrho_{2}I_{\left(No_{y}\right)} & * & *\\ {\bar{R}}_{♭}A_{♭}-\bar{\alpha }{\bar{R}}_{♭}K_{♭}C_{1}\; & {\bar{R}}_{♭}H\; & -\left(1-\bar{\alpha }\right){\bar{R}}_{♭}K_{♭}\; & -{\bar{R}}_{♭} & *\\ -\tilde{\alpha }{\bar{R}}_{♭}K_{♭}C_{2}\; & 0\; & \tilde{\alpha }{\bar{R}}_{♭}K_{♭}\; & 0 & -{\bar{R}}_{♭} \end{array}\right],\\ {ℶ}_{2,♭}≜ & \left[\begin{array}{ccccc} 0 & 0 & 0 & 0 & 0\\ 0 & 0 & 0 & 0 & 0\\ 0 & 0 & 0 & 0 & 0\\ {\bar{R}}_{♭}\Delta A & {\bar{R}}_{♭}\Delta H & 0 & 0 & 0\\ 0 & 0 & 0 & 0 & 0 \end{array}\right]. \end{array}$$

Noting that $\Delta A=\hat{J}\left(I_{2N}⊗J_{2}\right){\hat{J}}_{3A}$ and $\Delta H=\hat{J}\left(I_{2N}⊗J_{2}\right){\hat{J}}_{3H}$ from (6), one has ${ℶ}_{2,♭}={ℶ}_{21,♭}\left(I_{2N}⊗J_{2}\right){ℶ}_{22,♭}$ where

$$\begin{array}{cc} \; & \hat{J}≜\left[\begin{array}{cc} \bar{J} & 0\\ \bar{J} & 0 \end{array}\right],\bar{J}≜\mathrm{diag}\left\{{\bar{J}}_{11},{\bar{J}}_{12},\ldots ,{\bar{J}}_{1N}\right\},{\hat{J}}_{3A}≜\mathrm{diag}\left\{I_{\left(N\right)}⊗{\bar{J}}_{3A},0\right\},\\ \; & {\bar{J}}_{3A}≜\left[\begin{array}{ccc} J_{3A} & 0 & 0 \end{array}\right],{\bar{J}}_{1i}≜{\left[\begin{array}{ccc} J_{1i}^{T} & 0 & 0 \end{array}\right]}^{T},{\hat{J}}_{3H}≜\mathrm{diag}\left\{I_{\left(N\right)}⊗J_{3H},0\right\},\\ \; & {ℶ}_{21,♭}≜{\left[\begin{array}{cccc} 0 & 0 & 0 & {\left({\bar{R}}_{♭}\hat{J}\right)}^{T}0 \end{array}\right]}^{T},{ℶ}_{22,♭}≜\left[\begin{array}{ccccc} {\hat{J}}_{3A} & {\hat{J}}_{3H} & 0 & 0 & 0 \end{array}\right]. \end{array}$$

We have from (62) that

(63) $$\begin{array}{cc} \; & {ℶ}_{1,♭}+{ℶ}_{21,♭}\left(I_{2N}⊗J_{2}\right){ℶ}_{22,♭}+{\left({ℶ}_{21,♭}\left(I_{2N}⊗J_{2}\right){ℶ}_{22,♭}\right)}^{T}<0. \end{array}$$

Via utilizing the S-procedure Lemma and the Schur Complement Lemma, we yield that the following inequalities hold as long as (63) holds:

(64) $$\begin{array}{cc} \; & {ℶ}_{1,♭}+\hbar^{-1}{ℶ}_{21,♭}{ℶ}_{21,♭}^{T}+\hbar {ℶ}_{22,♭}^{T}{ℶ}_{22,♭}<0, \end{array}$$

(65) $$\begin{array}{cc} \; & \left[\begin{array}{ccc} {ℶ}_{1,♭} & * & *\\ {ℶ}_{21,♭}^{T} & -\hbar I & *\\ \hbar {ℶ}_{22,♭} & 0 & -\hbar I \end{array}\right]<0. \end{array}$$

Setting

(66) $$\begin{array}{c} {\bar{\Lambda }}_{♭}≜{\bar{R}}_{♭}K_{♭}, \end{array}$$

we see that (56) is the same as (65). Additionally, we notice from (51) that

(67) $$\begin{array}{cc} \Xi_{♭}= & \Xi_{1,♭}^{T}\left(I_{\left(3\right)}⊗{\bar{R}}_{♭}\right)\Xi_{1,♭}\\ = & \Xi_{1,♭}^{T}\left(I_{\left(3\right)}⊗{\bar{R}}_{♭}\right){\left(I_{\left(3\right)}⊗{\bar{R}}_{♭}\right)}^{-1}\left(I_{\left(3\right)}⊗{\bar{R}}_{♭}\right)\Xi_{1,♭}\\ = & \Xi_{2,♭}^{T}{\left(I_{\left(3\right)}⊗{\bar{R}}_{♭}\right)}^{-1}\Xi_{2,♭} \end{array}$$

with

$$\begin{array}{c} \Xi_{1,♭}≜\mathrm{diag}\{w_{0}E,\left(\theta \sqrt{\bar{\alpha }\left(1+\tau \right)}/2\right)K_{♭},\left(L\sqrt{\phi \bar{\alpha }\left(1+\tau^{-1}\right)}/\left(1-2\bar{\mu }\right)\right)K_{♭}\},\\ \Xi_{2,♭}≜\mathrm{diag}\{\;w_{0}{\bar{R}}_{♭}E,\left(\theta \sqrt{\bar{\alpha }\left(1+\tau \right)}/2\right){\bar{R}}_{♭}K_{♭},\left(L\sqrt{\phi \bar{\alpha }\left(1+\tau^{-1}\right)}/\left(1-2\bar{\mu }\right)\right){\bar{R}}_{♭}K_{♭}\}. \end{array}$$

Via adopting Schur Complement Lemma and combining (66), we see that (57) holds as long as the inequality below holds:

(68) $$\begin{array}{c} \Xi_{♭}<\Phi . \end{array}$$

In combination with (55) and (68), we see that

(69) $$\begin{array}{c} E\{∥\eta_{k}{∥}^{2}\}<\frac{\lambda_{\max}\left(R_{q_{0}}\right)}{\lambda_{\min}\left(R_{♭}\right)}{\left(1-\sigma \right)}^{k}{∥\eta_{0}∥}^{2}+\frac{\mathrm{tr}(\Phi )}{\sigma \lambda_{\min}\left(R_{♭}\right)}. \end{array}$$

From (69), one discovers that the ultimate upper bound of $E\{∥\eta_{k}{∥}^{2}\}$ is $\frac{\mathrm{tr}(\Phi )}{\sigma \lambda_{\min}\left(R_{♭}\right)}$, whose minimum is acquired by minimizing tr(Φ). The proof is then accomplished. □

**Remark** **2.** *Until now, the state and fault estimator design has been finished for a kind of nonlinear CNs utilizing BESs suffering from parameter uncertainties, RSCs, RODAs and noises. In Theorem 1, the estimator performance has been analyzed which ensures the exponential ultimate boundedness of the EED (33) in mean square, and the ultimate upper bound has been acquired. In Theorem 2, a sufficient condition has been presented by which the estimator (29) makes the EED (33) satisfy the performance constraint (34). The state and fault estimator (29) has been designed by tackling a minimized issue with matrix inequalities as constraint conditions, and a minimized ultimate upper bound has been yielded. In particular, Theorem 2 has involved all the crucial system parameters which include the total number of network nodes, the transition probability of switching couplings, the total number of coupling modes, the known matrices in parameter uncertainties, the statistical properties of process noise, parameters in the constraint conditions of nonlinearity and deception attack signal, the scope of measurement signal, the length of BBS, and the occurrence probabilities of bit flipping and deception attacks.*

**Remark** **3.** *In this paper, under an adequate consideration, the robust state and fault estimation has been realized for an uncertain CN employing BESs affected by nonlinearity, RSCs, RODAs and noises. In comparison with the existing estimation methods, merits of the robust state and fault estimation method developed in this paper lie in the following aspects: (1) the robust state and fault estimation issue is novel which contains BESs, RSCs, RODAs, parameter uncertainties and nonlinearity simultaneously; (2) the estimator model is novel which compensates the occurrence of deception attacks, describes the effect of bit flipping via constructing an equivalent stochastic noise, and estimates both the node state and fault; and (3) the developed state and fault estimation approach is novel in which the estimator gain is obtained by minimizing the ultimate upper bound with matrix inequality constraints.*

## 4. Tests and Results

In this section, two examples are conducted to test the effectiveness of the estimator design method in Theorem 2 with MATLAB software (edition 7.0.4) using performance index (34), which contain a numerical example and an application simulation example. Each example include estimator gain computation and curve plotting.

**Example** **1.** *Take account of a CN (N=4, U=3) with parameters shown in Appendix A.*

The nonlinear function $h_{x_{i,k}}$ is

$$\begin{array}{c} h_{x_{i,k}}=\left[\begin{array}{c} -0.6x_{i1,k}+0.3x_{i2,k}+\tanh\left(0.3x_{i1,k}\right)\\ 0.6x_{i2,k}-\tanh\left(0.2x_{i2,k}\right) \end{array}\right] \end{array}$$

where $x_{iℑ,k}$ (ℑ=1,2) denotes the *ℑ*-th component of $x_{i,k}$. We find that $\Psi_{1}=\left[\begin{array}{cc} -0.29 & 0.29\\ 0 & 0.6 \end{array}\right]$ and $\Psi_{2}=\left[\begin{array}{cc} -0.60 & 0.29\\ 0 & 0.4 \end{array}\right]$ fulfill (3).

The deception attack signal $d_{y_{i,k}}$ is

$$\begin{array}{c} d_{y_{i,k}}=\tanh\left(0.15y_{i,k}\right), \end{array}$$

and we see that (28) is satisfied with F=0.15.

By virtue of tackling the optimization issue (58), the estimator gains are obtained which are given in Appendix B.

Select the initial conditions $x_{1,0}={\left[\begin{array}{cc} 0.01 & 0.02 \end{array}\right]}^{T}$, $x_{2,0}={\left[\begin{array}{cc} -0.01 & 0.04 \end{array}\right]}^{T}$, $x_{3,0}={\left[\begin{array}{cc} 0.02 & 0.01 \end{array}\right]}^{T}$, $x_{4,0}={\left[\begin{array}{cc} 0.03 & 0.01 \end{array}\right]}^{T}$, ${\hat{x}}_{1,0}={\left[\begin{array}{cccc} 0.01 & 0.02 & 0 & 0 \end{array}\right]}^{T}$, ${\hat{x}}_{2,0}={\left[\begin{array}{cccc} -0.01 & 0.04 & 0 & 0 \end{array}\right]}^{T}$, ${\hat{x}}_{3,0}={\left[\begin{array}{cccc} 0.02 & 0.01 & 0 & 0 \end{array}\right]}^{T}$ and ${\hat{x}}_{4,0}={\left[\begin{array}{cccc} 0.03 & 0.01 & 0 & 0 \end{array}\right]}^{T}$. The unknown matrix is set as $J_{2}=\cos\left(3k\right)$. The fault signals are

$$\begin{array}{cc} \; & f_{1,k}=\left\{\begin{array}{cc} -\frac{k}{25}+1.5, & 0\leq k\leq 25,\\ 0.5, & \mathrm{else}, \end{array}\right.f_{4,k}=0.4\\ \; & f_{2,k}=\left\{\begin{array}{cc} \frac{k}{31}+0.4397, & 0\leq k\leq 19,\\ 1.0526, & 20\leq k\leq 30,\\ -\frac{k}{31}+2.0526, & \mathrm{else}, \end{array}\right.f_{3,k}=\left\{\begin{array}{cc} 0.5, & 0\leq k\leq 20,\\ -0.0214k+0.95, & 21\leq k\leq 35,\\ 0.2, & \mathrm{else}. \end{array}\right. \end{array}$$

**Figure 2:**
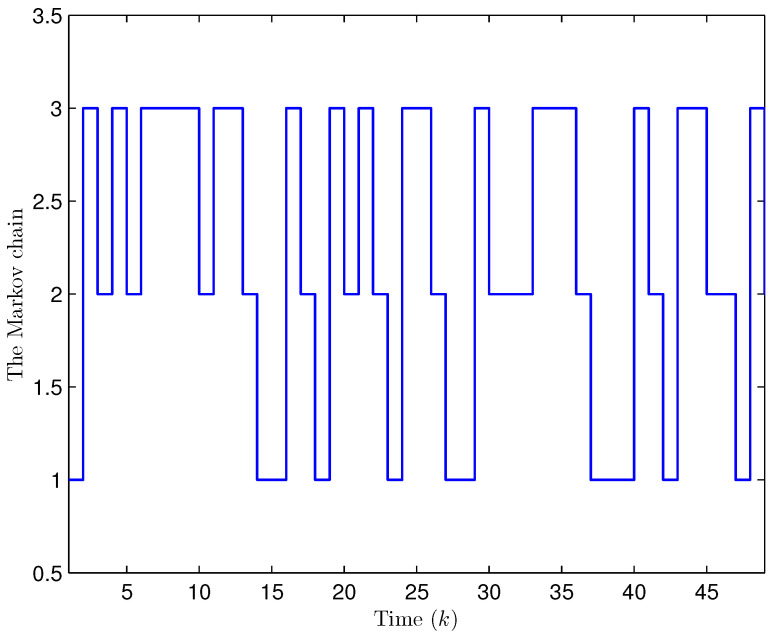
The switching of topologies and inner couplings (Example 1).

**Figure 3:**
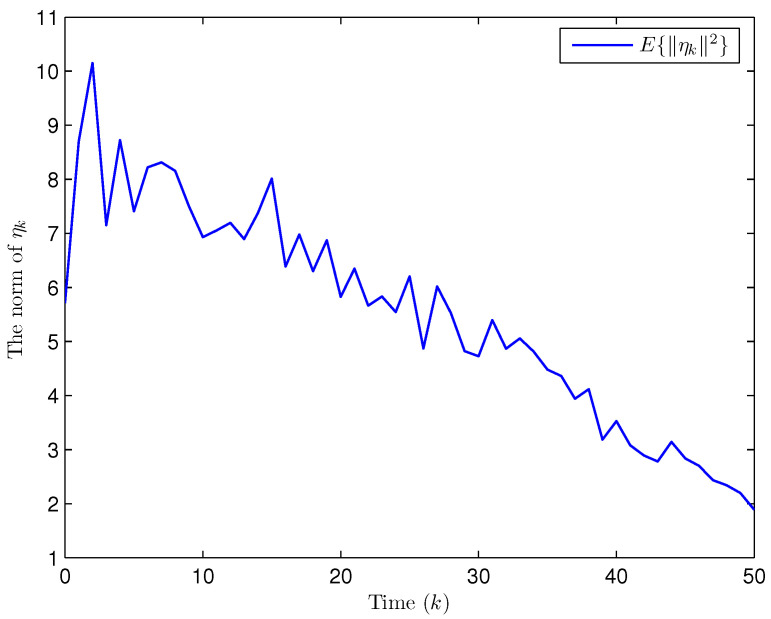
The expectation of the norm of the augmented EED (Example 1).

**Figure 4:**
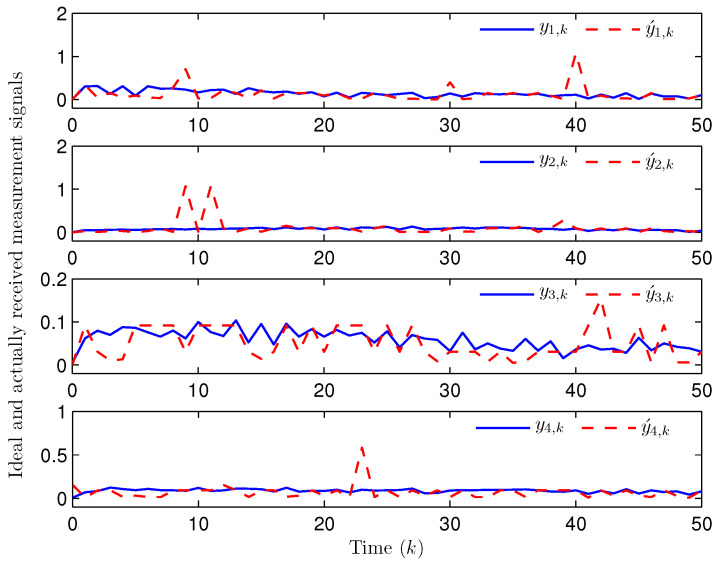
The ideal and actually received measurements (Example 1).

**Figure 5:**
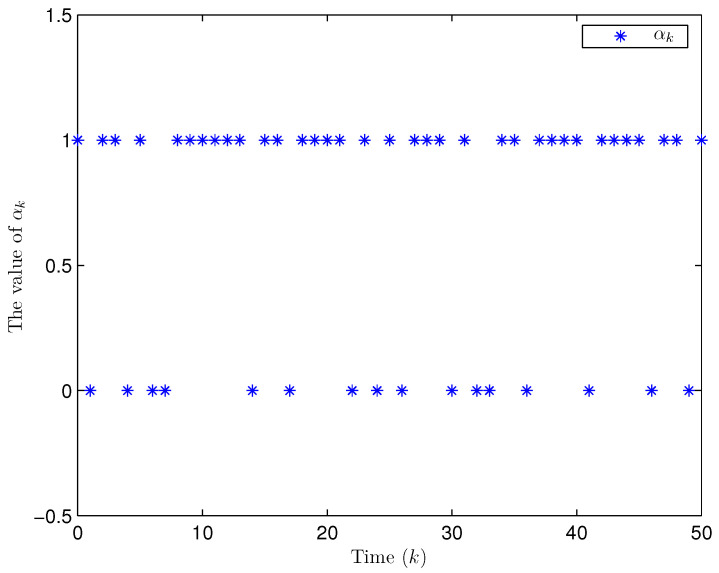
The random occurrence of deception attacks ($\alpha_{k}=0$) (Example 1).

**Figure 6:**
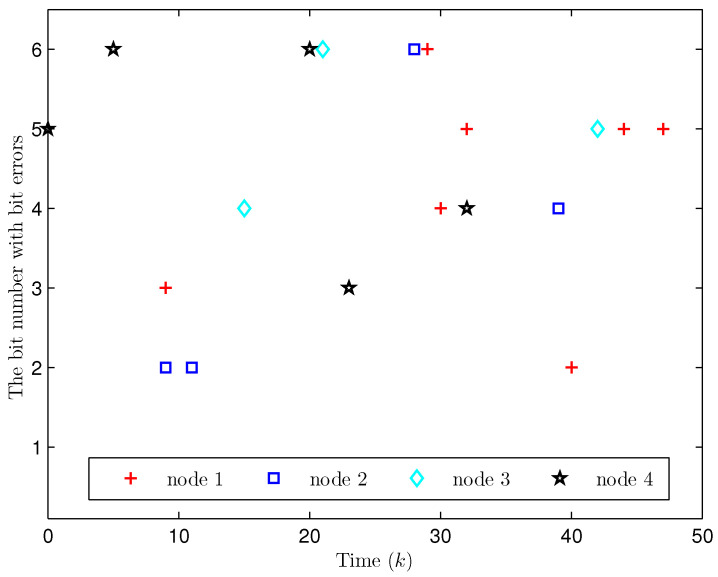
The random occurrence of bit errors in binary bits (Example 1).

**Figure 7:**
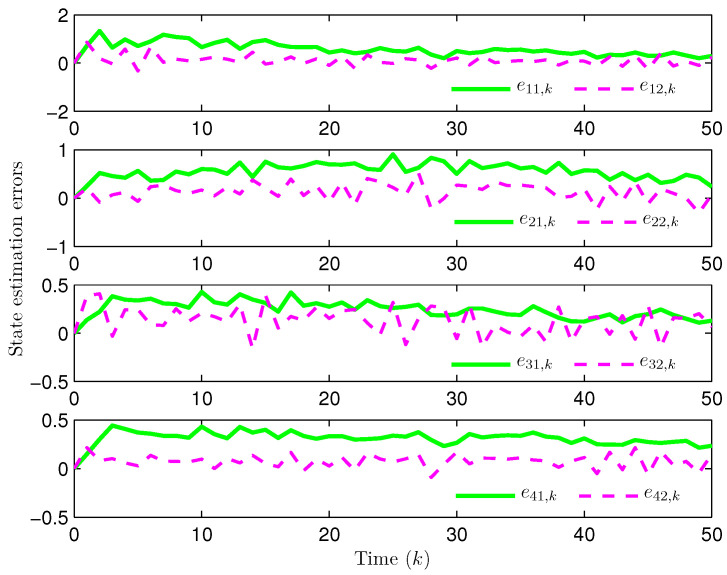
State estimation error (Example 1).

**Figure 8:**
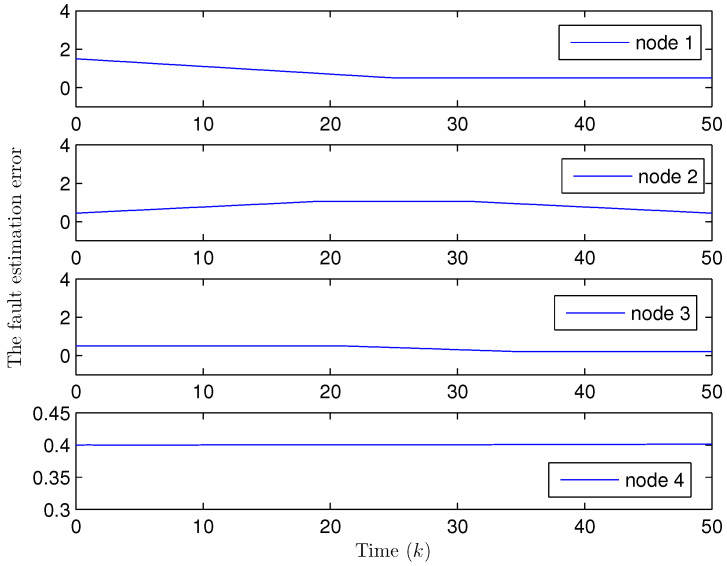
Fault estimation error (Example 1).

**Figure 9:**
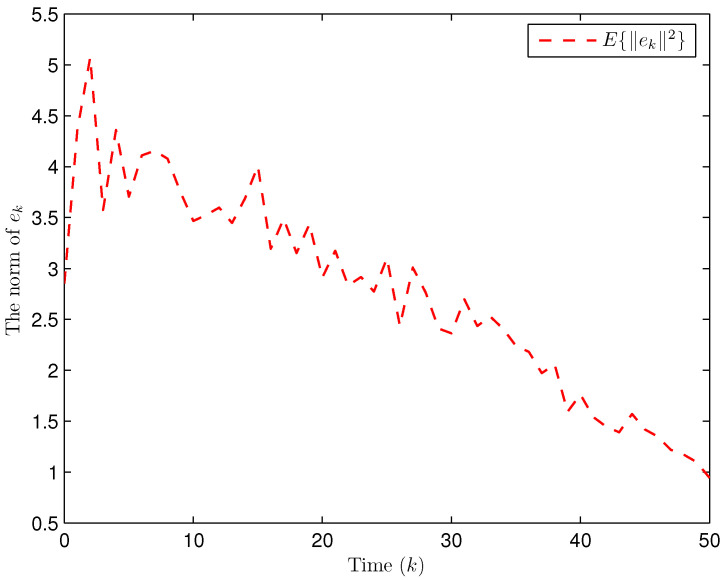
Expectation of norm of $e_{k}$ (Example 1).

**Figure 10:**
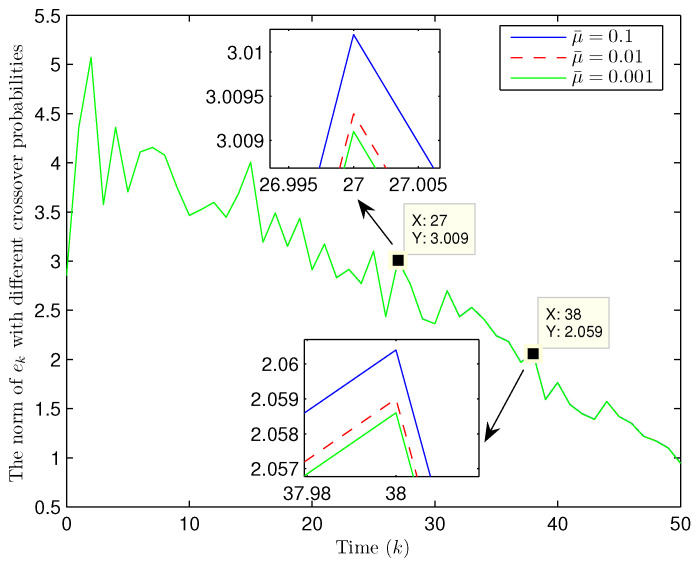
Norm of estimation error under different crossover probabilities (Example 1).

**Figure 11:**
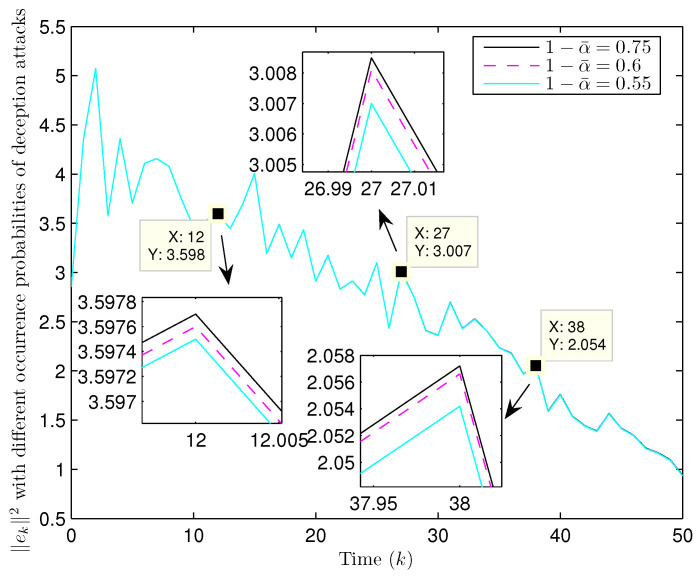
Norm of estimation error under different occurrence probabilities of deception attacks (Example 1).

**Figure 12:**
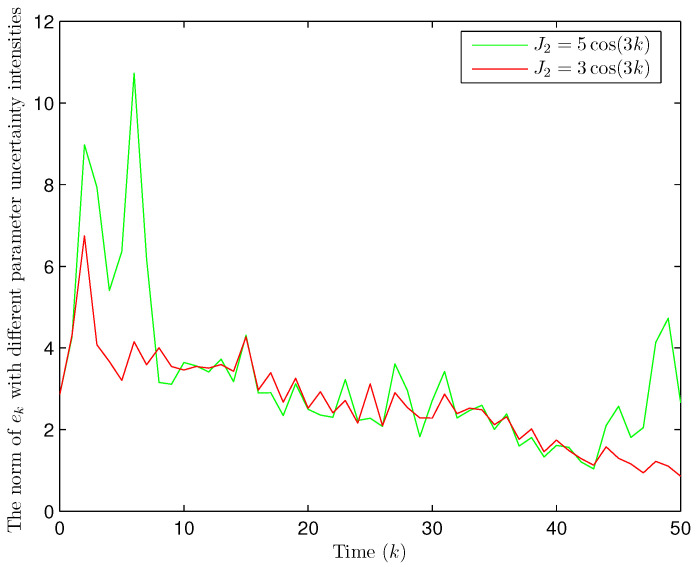
Norm of estimation error under different intensities of parameter uncertainties (Example 1).

**Figure 13:**
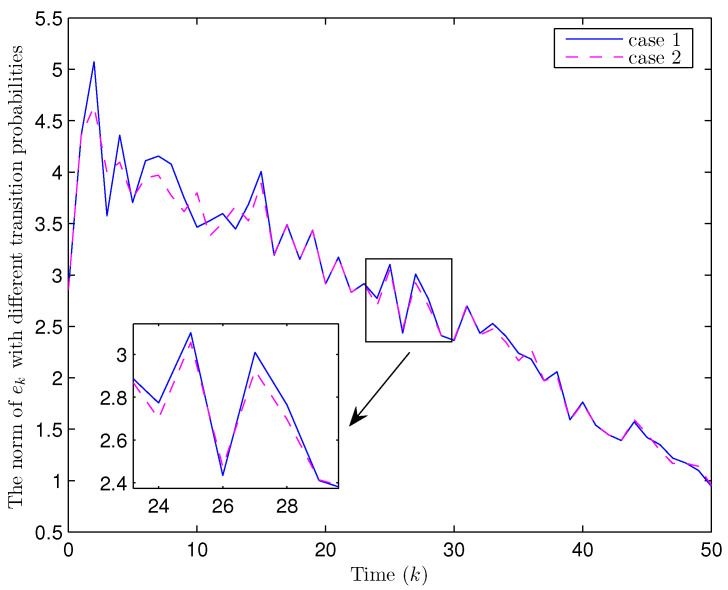
Norm of estimation error under different transition probabilities (Example 1).

Simulation curves are drawn in Figure 2, Figure 3, Figure 4, Figure 5, Figure 6, Figure 7, Figure 8, Figure 9, Figure 10, Figure 11, Figure 12 and Figure 13. Figure 2 represents the mode evolution of the Markov chain, i.e., the mode switching situation of the outer and inter couplings. Figure 3 is the expectation of the norm of the augmented EED (i.e., $E\{∥\eta_{k}{∥}^{2}\}$), which is bounded and illustrates that the exponential ultimate boundedness in mean square (34) is met. Figure 4 compares the original measurement signal and the received signal by the estimator. Figure 5 shows the value of $\alpha_{k}$, we see from (27) that when $\alpha_{k}=0$, deception attack appears. Figure 6 indicates the occurrence of random bit errors, for node 2, the 2nd bit occurs flipping at time steps 9 and 11, and the 6th bit and the 4th bit occur flipping at time steps 28 and 39. As is seen from Figure 5, $\alpha_{k}=1$ at time steps 9, 11, 28 and 39, that is, deception attacks do not occur at these time steps and the signal distortion is from bit flipping. In Figure 4, the differences size between $y_{2,k}$ and ${\overset{´}{y}}_{2,k}$ ($|{\overset{´}{y}}_{2,k}-y_{2,k}|$) are |1.071−0.06598|=1.00502, |1.071−0.07056|=1.00044, |0.0099−0.066|=0.0561 and |0.2755−0.05942|=0.21608 at time steps 9, 11, 28 and 39, respectively. We recognize from the above results that when the higher bit flips, there would be a bigger deviation value from the original signal, and this conforms to the fact that the higher bit has a bigger weight when conducting encoding and decoding. Estimation error curves of state and fault are drawn in Figure 7 and Figure 8, respectively. The evolution of expectation of the estimation error norm (i.e., $E\{∥e_{k}{∥}^{2}\}$) is plotted in Figure 9, which is not only bounded but also converged gradually with a range [0.9441,5.074]. Performance comparisons are conducted under several situations as follows. In Figure 10, the norm of the estimation error is presented with different crossover probabilities, which indicates that the bigger the crossover probability (the occurrence probability of bit flipping) is, the bigger the norm of the estimation error becomes. In Figure 11, the norm of the estimation error is exhibited with different occurrence probabilities of deception attacks, which reveals that the bigger the occurrence probability of deception attacks gets, the worse the estimation performance is. In Figure 12, the norm of the estimation error is given with different intensities of parameter uncertainties, which shows that the bigger the intensity of uncertainty turns, the bigger error between the estimate and the actual state and fault is obtained. In Figure 13, the norm of the estimation error is displayed with different transition probabilities of Markov chain (case 1: the transition probability in the parameter list of Example 1 and case 2: $\pi_{11}=0.1$, $\pi_{12}=0.45$$\pi_{13}=0.45$, $\pi_{21}=0.46$, $\pi_{22}=0.08$, $\pi_{23}=0.46$, $\pi_{31}=0.47$, $\pi_{32}=0.47$, $\pi_{33}=0.06$), which may suggest that the bigger the transition probability between different modes is (i.e., the bigger possibility of switching to another mode is), the bigger the resulted estimation error is.

**Example** **2.** *Take account of a CN constituted by interconnected flexible joint robots (N=5). For the i-th (i=1,2,…,5) robot, $x_{i1,k}$, $x_{i2,k}$, $x_{i3,k}$ and $x_{i4,k}$ indicate the angular rotation of the motor, the angular velocity of the motor, the angular rotation of the link, and the angular velocity of the link, respectively [61,62]. System parameters are listed in Appendix C, and other parameters are the same as those in Example 1.*

The nonlinear function is presented as

$$\begin{array}{c} h_{x_{i,k}}={\left[\begin{array}{cccc} 0 & 0 & 0 & -3.33\sin(x_{i3,k}) \end{array}\right]}^{T} \end{array}$$

where $x_{i3,k}$ is the third element of $x_{i,k}$, and we see that $\Psi_{1}=\left[\begin{array}{cccc} 0 & 0 & 0 & 0\\ 0 & 0 & 0 & 0\\ 0 & 0 & 0 & 0\\ 0 & 0 & -6 & 0 \end{array}\right]$ and $\Psi_{2}=\left[\begin{array}{cccc} 0 & 0 & 0 & 0\\ 0 & 0 & 0 & 0\\ 0 & 0 & 0 & 0\\ 0 & 0 & 6 & 0 \end{array}\right]$ meet condition (3).

Via solving the optimization issue (58), gains of estimator (29) are obtained as Appendix D shows.

Select the initial states of the system (1) and the estimator (29) as

$$\begin{array}{cc} \; & x_{1,0}={\left[\begin{array}{cccc} 0.01 & 0.02 & 0.05 & 0.02 \end{array}\right]}^{T},x_{2,0}={\left[\begin{array}{cccc} -0.01 & 0.04 & -0.03 & 0.02 \end{array}\right]}^{T},\\ \; & x_{3,0}={\left[\begin{array}{cccc} 0.02 & 0.01 & 0.01 & 0.02 \end{array}\right]}^{T},x_{4,0}={\left[\begin{array}{cccc} 0.03 & 0.01 & 0.02 & 0.03 \end{array}\right]}^{T},\\ \; & x_{5,0}={\left[\begin{array}{cccc} 0.02 & 0.04 & 0.03 & 0.01 \end{array}\right]}^{T},{\hat{x}}_{1,0}={\left[\begin{array}{cccccc} 0.01 & 0.02 & 0.02 & 0.01 & 0 & 0 \end{array}\right]}^{T},\\ \; & {\hat{x}}_{2,0}={\left[\begin{array}{cccccc} -0.01 & 0.04 & 0.03 & 0.05 & 0 & 0 \end{array}\right]}^{T},{\hat{x}}_{3,0}={\left[\begin{array}{cccccc} 0.02 & 0.01 & 0.04 & 0.06 & 0 & 0 \end{array}\right]}^{T},\\ \; & {\hat{x}}_{4,0}={\left[\begin{array}{cccccc} 0.03 & 0.01 & 0.01 & 0.03 & 0 & 0 \end{array}\right]}^{T},{\hat{x}}_{5,0}={\left[\begin{array}{cccccc} 0.02 & 0.02 & -0.01 & 0.02 & 0 & 0 \end{array}\right]}^{T}. \end{array}$$

**Figure 14:**
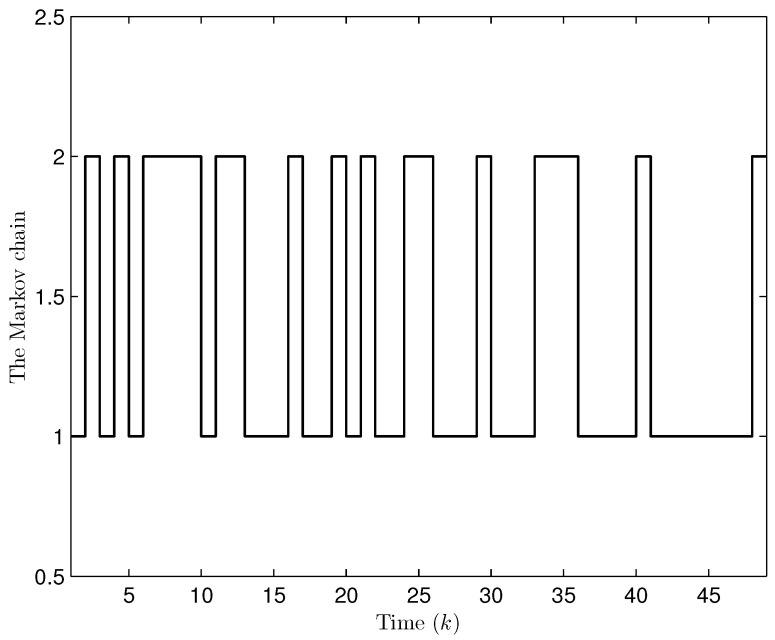
The switching of topologies and inner couplings (Example 2).

**Figure 15:**
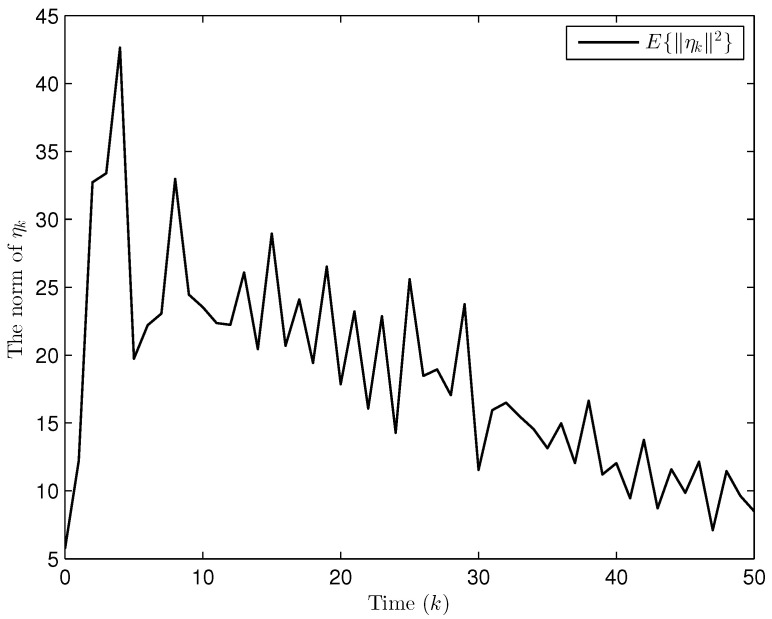
Evolution of the expectation of the norm of $\eta_{k}$ (Example 2).

**Figure 16:**
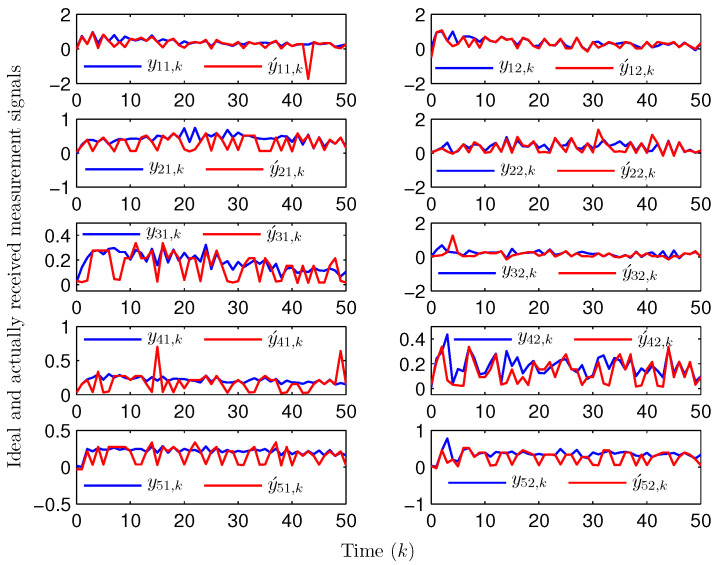
The ideal and actually received measurements (Example 2).

**Figure 17:**
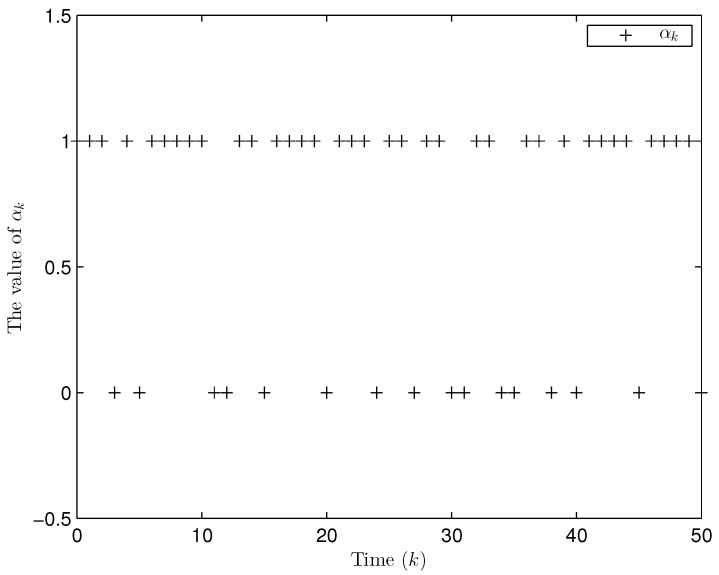
The occurrence of deception attacks ($\alpha_{k}=0$) (Example 2).

**Figure 18:**
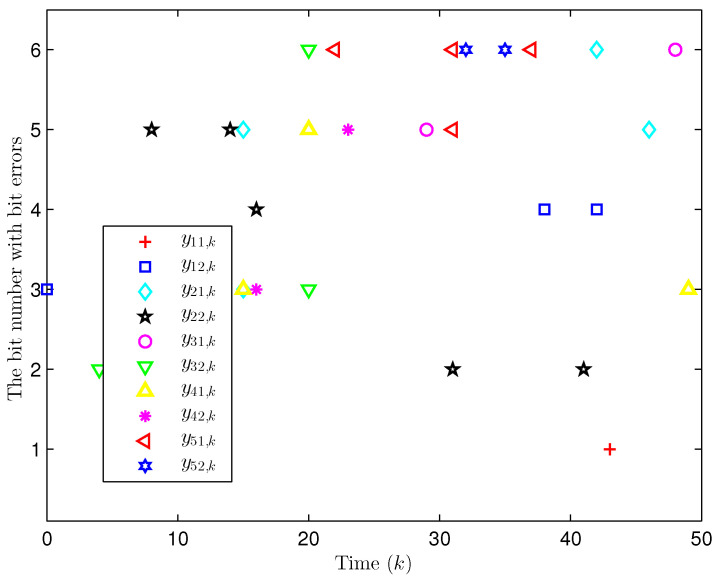
The random occurrence of bit flipping (Example 2).

Apart from fault signals in Example 1, the fault signal of the 5th robot is $f_{5,k}=0.6$. Simulation curves are drawn in Figure 14, Figure 15, Figure 16, Figure 17 and Figure 18. Figure 14 reveals the mode switching of outer and inner couplings characterized by a Markov chain. Figure 15 reveals that $E\{∥\eta_{k}{∥}^{2}\}$ is bounded, i.e., the performance constraint (34) is satisfied with the designed estimator (29). Figure 16 shows that, for each network node, there exist some differences between the original measurement signals and the ones actually attained by the estimator, which reflect the impact from quantization error, bit flipping and deception attack possibly. Figure 17 illustrates the random occurrence situation of deception attacks, which means that deception attacks occur when $\alpha_{k}=0$ according to (27). Figure 18 demonstrates the occurrence situation of bit flipping for measurement components of 5 nodes, respectively. Simulation results testify that the designed estimator (29) is capable of estimating node state and fault of CN (1) correctly and effectively.

## 5. Conclusions

This paper has designed the state and fault estimator for a class of nonlinear CNs subject to parameter uncertainties, stochastic switching topologies and inner couplings, RODAs, and bounded stochastic noises using BESs. Fault has been taken account of in the network node state whose second-order difference is zero. A norm-bounded multiplicative form has been adopted to depict parameter uncertainties, and a Markov chain has been employed to characterize the random switching phenomena of outer and inner couplings. When utilizing BESs, a probabilistic quantizer has been employed to encode the measurement signal into a BBS, and random bit flipping has been concerned whose effect is regarded as a constructed stochastic noise after statistical property analysis. The random occurrences of bit flipping and deception attacks have been reflected via two sets of Bernoulli-distributed random variables. By virtue of Lyapunov stability theory and matrix inequality method, a sufficient condition has been yielded that assures that the state and fault EED is exponentially ultimately bounded in mean square, and a minimized ultimate upper bound is attained. Gains of the estimator have been attained by solving the solution to a minimized issue constrained by matrix inequalities. The simulation results have testified that the developed estimation method is accurate and valid. In future, the authors intend to (1) extend the acquired security-guaranteed estimation method to sensor networks [63] and circuit systems [64] and (2) design estimators using a stochastic scheduling protocol [65] and a relay channel [66].

## Data Availability

The original contributions presented in this study are included in the article. Further inquiries can be directed to the corresponding author.

## Abbreviations

The following abbreviations are used in this manuscript:

CN	Complex network
BBS	Binary bit string
BES	Binary encoding scheme
BSC	Binary symmetric channel
RODAs	Randomly occurring deception attacks
RSCs	Randomly switching couplings
EED	Estimation error dynamics

## Appendix A

$$\begin{array}{cc} \; & A_{1}=\left[\begin{array}{cc} 0.45 & 0.27\\ -0.51 & -0.5 \end{array}\right],A_{2}=\left[\begin{array}{cc} 0.32 & 0.2\\ -0.61 & -0.3 \end{array}\right],A_{3}=\left[\begin{array}{cc} 0.47 & -0.1\\ -0.29 & -0.4 \end{array}\right],A_{4}=\left[\begin{array}{cc} 0.5 & 0.18\\ -0.49 & -0.36 \end{array}\right],\\ \; & H_{1}=0.22I_{\left(2\right)},H_{2}=0.3I_{\left(2\right)},H_{3}=0.14I_{\left(2\right)},H_{4}=0.4I_{\left(2\right)},J_{11}=J_{12}=J_{13}=J_{14}=I_{\left(2\right)},\\ \; & J_{3A}=\left[\begin{array}{cc} 0.1 & 0.1\\ 0.1 & 0.1 \end{array}\right],J_{3H}=\left[\begin{array}{cc} 0.08 & 0.08\\ 0.08 & 0.08 \end{array}\right],E_{1}=\left[\begin{array}{c} 0.13\\ 0.15 \end{array}\right],E_{2}=\left[\begin{array}{c} -0.11\\ 0.25 \end{array}\right],E_{3}=\left[\begin{array}{c} 0.04\\ -0.17 \end{array}\right],\\ \; & P_{1}=\left[\begin{array}{cccc} -0.46 & 0.23 & 0 & 0.23\\ 0.23 & -0.46 & 0.23 & 0\\ 0 & 0.23 & -0.46 & 0.23\\ 0.23 & 0 & 0.23 & -0.46 \end{array}\right],P_{2}=\left[\begin{array}{cccc} -0.78 & 0.27 & 0.26 & 0.25\\ 0.27 & -0.4 & 0.08 & 0.05\\ 0.26 & 0.08 & -0.6 & 0.26\\ 0.25 & 0.05 & 0.26 & -0.56 \end{array}\right],\\ \; & P_{3}=\left[\begin{array}{cccc} -0.063 & 0.021 & 0.018 & 0.024\\ 0.021 & -0.08 & 0.035 & 0.024\\ 0.018 & 0.035 & -0.072 & 0.019\\ 0.024 & 0.024 & 0.019 & -0.067 \end{array}\right],Y=\left[\begin{array}{ccc} 0.5 & 0.38 & 0.12\\ 0.6 & 0.26 & 0.14\\ 0.29 & 0.3 & 0.41 \end{array}\right],E_{4}=\left[\begin{array}{c} 0.03\\ 0.09 \end{array}\right],\\ \; & \Gamma_{1}=\mathrm{diag}\left\{0.3,0.4\right\},\Gamma_{2}=\mathrm{diag}\left\{0.7,0.5\right\},\Gamma_{3}=\mathrm{diag}\left\{0.5,0.2\right\},w_{0}=0.58,C_{1}=\left[\begin{array}{cc} 0.21 & 0.18 \end{array}\right],\\ \; & G_{1}=\left[\begin{array}{c} 0.5\\ 0.6 \end{array}\right],G_{2}=\left[\begin{array}{c} 0.52\\ 0.62 \end{array}\right],G_{3}=\left[\begin{array}{c} 0.3\\ 0.7 \end{array}\right],G_{4}=\left[\begin{array}{c} 0.4\\ 0.6 \end{array}\right],C_{2}=\left[\begin{array}{cc} 0.11 & 0.12 \end{array}\right],C_{3}=\left[\begin{array}{cc} 0.19 & 0.09 \end{array}\right],\\ \; & C_{4}=\left[\begin{array}{cc} 0.24 & 0.16 \end{array}\right],\bar{\mu }=0.01,S=6,\theta =0.06,\bar{\alpha }=0.7,\sigma =0.2,\tau =0.5. \end{array}$$

## Appendix B

$$\begin{array}{cc} \; & K_{1,1}=10^{-8}\times \left[\begin{array}{c} -0.0261\\ -0.0815\\ -0.2225\\ 0.2276 \end{array}\right],K_{2,1}=10^{-4}\times \left[\begin{array}{c} -0.1415\\ -0.9985\\ -0.2085\\ 0.3032 \end{array}\right],K_{3,1}=10^{-4}\times \left[\begin{array}{c} -0.0716\\ -0.6205\\ -0.0753\\ 0.3802 \end{array}\right],\\ \; & K_{4,1}=10^{-4}\times \left[\begin{array}{c} 0.0337\\ -0.2096\\ -0.1162\\ 0.2656 \end{array}\right],K_{1,2}=10^{-5}\times \left[\begin{array}{c} -0.0402\\ -0.0610\\ -0.4784\\ -0.6030 \end{array}\right],K_{2,2}=10^{-5}\times \left[\begin{array}{c} -0.0413\\ -0.1682\\ -0.1706\\ -0.1965 \end{array}\right],\\ \; & K_{3,2}=10^{-6}\times \left[\begin{array}{c} -0.2727\\ -0.5433\\ -0.4411\\ -0.6975 \end{array}\right],K_{4,2}=10^{-6}\times \left[\begin{array}{c} -0.1786\\ -0.9122\\ -0.5398\\ -0.4430 \end{array}\right],K_{1,3}=10^{-7}\times \left[\begin{array}{c} 0.1933\\ 0.0516\\ 0.1742\\ 0.3104 \end{array}\right],\\ \; & K_{2,3}=10^{-7}\times \left[\begin{array}{c} -0.1674\\ -0.0436\\ -0.1184\\ -0.2130 \end{array}\right],K_{3,3}=10^{-7}\times \left[\begin{array}{c} -0.0688\\ -0.0165\\ 0.0711\\ 0.5633 \end{array}\right],K_{4,3}=10^{-4}\times \left[\begin{array}{c} -0.0084\\ 0.0007\\ 0.0041\\ -0.3924 \end{array}\right]. \end{array}$$

## Appendix C

$$\begin{array}{cc} \; & A_{i}=\left[\begin{array}{cccc} -0.0732 & 0.0002 & 0.2089 & -0.0241\\ -0.4871 & -0.0756 & 1.3048 & 0.2089\\ -0.0368 & -0.0098 & 0.2354 & 0.0560\\ 1.5869 & 0.0851 & -3.4860 & 0.2354 \end{array}\right],G_{1}=\left[\begin{array}{c} 0.5\\ 0.6\\ 0.5\\ 0.6 \end{array}\right],G_{2}=\left[\begin{array}{c} 0.52\\ 0.62\\ 0.4\\ 0.32 \end{array}\right],G_{3}=\left[\begin{array}{c} 0.3\\ 0.7\\ 0.4\\ 0.2 \end{array}\right],\\ \; & H_{i}=I_{\left(4\right)},J_{1i}=I_{\left(4\right)},C_{i}=\left[\begin{array}{cccc} 1 & 0 & 0 & 0\\ 0 & 1 & 0 & 0 \end{array}\right]\left(i=1,2,\ldots ,5\right),U=2,\Gamma_{1}=\mathrm{diag}\left\{0.3,0.4,0.3,0.4\right\},\\ \; & P_{1}=\left[\begin{array}{ccccc} -0.69 & 0.23 & 0 & 0.23 & 0.23\\ 0.23 & -0.76 & 0.23 & 0 & 0.3\\ 0 & 0.23 & -0.68 & 0.23 & 0.22\\ 0.23 & 0 & 0.23 & -0.57 & 0.11\\ 0.23 & 0.3 & 0.22 & 0.11 & -0.86 \end{array}\right],G_{4}=\left[\begin{array}{c} 0.4\\ 0.6\\ 0.3\\ 0.21 \end{array}\right],G_{5}=\left[\begin{array}{c} 0.24\\ 0.76\\ 0.3\\ 0.43 \end{array}\right],E_{1}=\left[\begin{array}{c} 0.13\\ 0.15\\ 0.13\\ 0.15 \end{array}\right],\\ \; & P_{2}=\left[\begin{array}{ccccc} -0.78 & 0.14 & 0.26 & 0.25 & 0.13\\ 0.27 & -0.6 & 0.08 & 0.05 & 0.2\\ 0.26 & 0.08 & -0.74 & 0.26 & 0.14\\ 0.25 & 0.05 & 0.26 & -0.63 & 0.07\\ 0.13 & 0.2 & 0.14 & 0.07 & -0.54 \end{array}\right],E_{2}=\left[\begin{array}{c} -0.11\\ 0.25\\ -0.11\\ 0.25 \end{array}\right],E_{5}=\left[\begin{array}{c} 0.02\\ 0.07\\ 0.03\\ 0.04 \end{array}\right],E_{4}=\left[\begin{array}{c} 0.03\\ 0.09\\ 0.03\\ 0.09 \end{array}\right],\\ \; & \Gamma_{2}=\mathrm{diag}\left\{0.7,0.5,0.7,0.5\right\},\pi_{11}=0.62,\pi_{12}=0.38,\pi_{21}=0.47,\pi_{22}=0.53,F=0.15I_{\left(2\right)},\\ \; & E_{3}=\left[\begin{array}{c} 0.04\\ -0.17\\ 0.04\\ -0.17 \end{array}\right],J_{3A}=\left[\begin{array}{cccc} 0.03 & 0.03 & 0.03 & 0.03\\ 0.03 & 0.03 & 0.03 & 0.03\\ 0.03 & 0.03 & 0.03 & 0.03\\ 0.03 & 0.03 & 0.03 & 0.03 \end{array}\right],J_{3H}=\left[\begin{array}{cccc} 0.08 & 0.08 & 0.08 & 0.08\\ 0.08 & 0.08 & 0.08 & 0.08\\ 0.08 & 0.08 & 0.08 & 0.08\\ 0.08 & 0.08 & 0.08 & 0.08 \end{array}\right] \end{array}$$

## Appendix D

$$\begin{array}{cc} \; & K_{1,1}=10^{-4}\times \left[\begin{array}{cc} -5.2623 & -4.6744\\ 1.6105 & -12.1112\\ -0.0018 & -0.0127\\ 0.0047 & 0.0019\\ 0.0015 & -0.0023\\ -0.0035 & -0.0043 \end{array}\right],K_{2,1}=10^{-6}\times \left[\begin{array}{cc} -0.0173 & -0.0457\\ 0.1927 & -0.3896\\ -0.5113 & -0.5015\\ 0.5984 & -0.0996\\ 0.1365 & -0.3824\\ -0.2985 & -0.3535 \end{array}\right],\\ \; & K_{3,1}=10^{-6}\times \left[\begin{array}{cc} 0.1539 & -0.0730\\ 0.2981 & -0.3791\\ -0.3335 & -0.4556\\ 0.5928 & -0.1173\\ 0.1019 & -0.3056\\ -0.3539 & -0.3918 \end{array}\right],K_{4,1}=10^{-6}\times \left[\begin{array}{cc} 0.1268 & -0.1046\\ 0.2940 & -0.3775\\ -0.3155 & -0.4157\\ 0.5856 & -0.1278\\ 0.1496 & -0.1506\\ -0.1622 & -0.1068 \end{array}\right],\\ \; & K_{5,1}=10^{-6}\times \left[\begin{array}{cc} -0.1767 & -0.0840\\ 0.0298 & -0.3218\\ -0.1220 & -0.3142\\ 0.3366 & -0.2064\\ 0.0866 & -0.0902\\ -0.3273 & -0.0725 \end{array}\right],K_{1,2}=10^{-7}\times \left[\begin{array}{cc} 5.0509 & 11.7985\\ 0.3133 & 2.0184\\ 0.0028 & 0.0066\\ -0.0104 & -0.0197\\ 0.0030 & 0.0052\\ 0.0004 & 0.0071 \end{array}\right], \end{array}$$

$$\begin{array}{cc} \; & K_{2,2}=10^{-8}\times \left[\begin{array}{cc} 0.0106 & 0.0154\\ 0.0024 & 0.0036\\ 0.0153 & 0.0194\\ -0.1155 & -0.1437\\ 0.0182 & 0.0240\\ 0.0204 & 0.0255 \end{array}\right],K_{3,2}=10^{-8}\times \left[\begin{array}{cc} 0.0106 & 0.0171\\ 0.0021 & 0.0040\\ 0.0162 & 0.0230\\ -0.0945 & -0.1311\\ 0.0178 & 0.0262\\ 0.0212 & 0.0307 \end{array}\right],\\ \; & K_{4,2}=10^{-7}\times \left[\begin{array}{cc} 0.0016 & 0.0016\\ -0.0004 & 0.0006\\ 0.0014 & 0.0031\\ -0.0167 & -0.0113\\ -0.3486 & 0.0924\\ 2.1322 & -0.5944 \end{array}\right],K_{5,2}=10^{-6}\times \left[\begin{array}{cc} 0.0027 & -0.0003\\ 0.0029 & 0.0025\\ -0.0012 & -0.0005\\ -0.0096 & -0.0007\\ -0.0933 & 0.1384\\ 0.3107 & -0.2065 \end{array}\right]. \end{array}$$
